# Combinatorial targeting of MTHFD2 and PAICS in purine synthesis as a novel therapeutic strategy

**DOI:** 10.1038/s41419-019-2033-z

**Published:** 2019-10-17

**Authors:** Chantal Hoi Yin Cheung, Chia-Lang Hsu, Chao-Yin Tsuei, Tzu-Ting Kuo, Chen-Tsung Huang, Wen-Ming Hsu, Yun-Hsien Chung, Hsin-Yi Wu, Cheng-Chih Hsu, Hsuan-Cheng Huang, Hsueh-Fen Juan

**Affiliations:** 1 Department of Life Science, National Taiwan University, Taipei, 10617 Taiwan; 2 Department of Medical Research, National Taiwan University Hospital, Taipei, 10002 Taiwan; 3 Institute of Molecular and Cellular Biology, National Taiwan University, Taipei, 10617 Taiwan; 4 Graduate Institute of Biomedical Electronics and Bioinformatics, National Taiwan University, Taipei, 10617 Taiwan; 5 Department of Surgery, National Taiwan University Hospital and College of Medicine National Taiwan University, Taipei, 10017 Taiwan; 6 Department of Chemistry, National Taiwan University, Taipei, 10617 Taiwan; 7 Institute of Biomedical Informatics, National Yang-Ming University, Taipei, 11221 Taiwan

**Keywords:** Metabolomics, Paediatric cancer, Cell growth, Target identification, Transcriptomics

## Abstract

*MYCN*-amplified (MNA) neuroblastoma is an aggressive neural crest-derived pediatric cancer. However, MYCN is indispensable for development and transcriptionally regulates extensive network of genes. Integrating anti-MYCN ChIP-seq and gene expression profiles of neuroblastoma patients revealed the metabolic enzymes, *MTHFD2* and *PAICS*, required for one-carbon metabolism and purine biosynthesis were concomitantly upregulated, which were more susceptible to metastatic neuroblastoma. Moreover, we found that MYCN mediated the folate cycle via MTHFD2, which contributed one-carbon unit to enhance purine synthesis, and further regulated nucleotide production by PAICS in response to cancer progression. Dual knockdown of the MYCN-targeted gene pair, *MTHFD2* and *PAICS*, in MNA neuroblastoma cells synergically reduced cell proliferation, colony formation, migration ability, and DNA synthesis. By systematically screening the compound perturbagens, the gene expression levels of *MTHFD2* and *PAICS* were specifically suppressed by anisomycin and apicidin across cell lines, and our co-treatment results also displayed synergistic inhibition of MNA neuroblastoma cell proliferation. Collectively, targeting a combination of MYCN-targeted genes that interrupts the interconnection of metabolic pathways may overcome drug toxicity and improve the efficacy of current therapeutic agents in MNA neuroblastoma.

## Introduction

Neuroblastoma is an embryonal tumor derived from the sympathetic nervous system and accounts for 15% of pediatric cancer mortality^1,2^. Although the outcome of high-risk patients has improved on the use of therapy, the majority eventually relapses and develops resistance with a 5-year event-free survival <50%^3,4^. High-risk (HR) neuroblastoma is characterized by V-myc avian myelocytomatosis viral oncogene neuroblastoma derived homolog (*MYCN)* amplification and segmental DNA copy number aberrations which are strongly correlated with poor prognosis, metastasis, and even treatment failure^5–9^. MYCN is a transcription factor that governs downstream signaling pathways for a broad range of fundamental processes during embryogenesis and development^10,11^. Amplification of *MYCN* is associated with tumor initiation and progression, and occurs in ~20% of all neuroblastoma cases, with a higher incidence in INSS stages 3 and 4 compared to stages 1, 2, and 4S, suggesting a contribution in regulating oncogenic pathways^3,12,13^.

Alteration of cellular metabolism to maintain energy status for rapid cell progression has been considered as a feature of cancer cells^14,15^. MYCN has been closely tied to the regulation of neuroblastoma cell growth, and confers the serine-glycine-one-carbon pathway to promote metabolic reprogramming in HR neuroblastoma^16,17^. *MYCN*-amplified (MNA) neuroblastoma increases cell growth by glutamine transport and metabolism enhancement, thus depletion of glutamine causes the limitation of TCA cycle intermediates and eventually cell death^18^. Moreover, MYCN has been shown to stimulate mitochondrial biogenesis of β-oxidation and glucose metabolism in cancer progression^19,20^.

Cellular metabolism relies on highly regulated sequential enzyme-catalyzed reactions to acquire the essential components such as purines to generate DNA and RNA molecules^21^. Other than nucleotide formation, purines are the constituents of important biomolecules as energy and signal transduction cofactors to drive cellular processes^22^. Interestingly, a co-localization of purinosomes with mitochondria was found under purine-depleted environment suggesting a functional interaction between mitochondria and purine biosynthesis^23^. The mitochondrial folate enzyme methylenetetrahydrofolate dehydrogenase 2 (*MTHFD2*) is the most consistently overexpressed metabolic gene in cancer which plays a pivotal role in regulating the release of formate into cytoplasm^24^. In addition, serine is a precursor for the synthesis of glycine in one carbon pool by folate pathway at the mitochondria compartment which is further used up for purine biosynthesis^25^.

Collectively, we aimed to explore the relationship of MYCN-mediated pathways by integrating ChIP-seq and gene expression profiles to comprehensively dissect potential MYCN-targeted genes. Here, we elucidated that MNA neuroblastoma favors one carbon metabolism and purine biosynthesis by the up-regulation of MYCN-targeted metabolic genes *MTHFD2* and *PAICS*. Our results also indicated that dual knockdown of MTHFD2/PAICS significantly reduced cell proliferation, colony formation, and migration which might diminish the aggressiveness and tumor progression ability in MNA neuroblastoma. Furthermore, combining the compound perturbagens to MYCN target gene pair displayed synergistic effects in MNA neuroblastoma cells which provides therapeutic opportunities for children with neuroblastoma.

## Materials and methods

### Differential expression analysis

Microarray datasets of neuroblastoma samples were acquired from NCBI Gene Expression Omnibus (GEO) with accession number GSE45547, GSE3446, GSE19274, GSE16254, GSE12460, and GSE16237, including 1065 neuroblastoma. For multiple probes corresponding to a single gene, median expression value of those probes was taken. Total 11,939 common genes of six datasets were considered for downstream analysis. The expression values were log transformed and normalized by quantile normalization across all samples. HR neuroblastoma (age at diagnosis >18 months and stage 4) were classified into HR-MNA (*n* = 67) and HR-non-MNA (*n* = 156) groups based on the *MYCN* status. Significance Analysis of Microarrays (SAM) was used to identify differentially expressed genes between HR-MNA and HR-non-MNA with false discovery rate (FDR) <0.001^26^.

### Public data sources and bioinformatics analysis

MYCN-bound genes were obtained from our previous work^27^ which ChIP-seq was used for genome-wide identification of MYCN regulatory networks. Two independent neuroblastma cohorts (SEQC and TARGET) were used for survival and correlation analyses. SEQC cohort was download from GEO with accession number GSE47792 and TARGET cohort was queried via GDC data portal (https://portal.gdc.cancer.gov/). The H3K4me3 and H3K27ac epigenetic profiles were obtained from ENCODE project. KEGG enrichment analysis was performed using the R/Bioconductor package clusterProfiler^28^.

### Cell culture

Human neuroblastoma cell lines SK-N-DZ (CRL-2149), SK-N-SH (HTB-11), SK-N-BE(2)-C (CRL-2268) were obtained from ATCC. SH-SY5Y, SK-N-AS, and SK-N-FI neuroblastoma cell lines were obtained from Dr. Yung-Feng Liao (Academia Sinica, Taipei, Taiwan). The conditional *MYCN*-expressing SHEP Tet21N cell line (RRID, CVCL_9812) was cultured in the presence of 1 μg/ml of tetracycline (Sigma) for 48 h and the Tet21N control cells were cultured in the presence of 1 μg/ml of 70% ethanol. All cell lines were maintained in Dulbecco’s modified Eagle’s medium (Gibco) with 10% fetal bovine serum (Biological Industries) at 37 °C in a humidified incubator with 5% CO_2_.

### RNA isolation of neuroblastoma cells and patient tissues, and cDNA synthesis

Neuroblastoma cells and tissue samples were homogenized by TRIzol reagent (Invitrogen). Total RNA of cells and tissue samples were prepared by Direct-zol™ RNA MiniPrep kit (ZYMO RESEARCH). RNA concentration and quality were determined by NanoDrop ND-1000 (NanoDrop Technologies). In all, 100 ng of total RNA of each tissue sample or 500 ng of total RNA template of each neuroblastoma cell line sample was reverse transcript to cDNA by using RevertAidTM H Minus First Strand cDNA Synthesis Kit (Thermo Scientific) with oligo (dT) 18 primer. The cDNA samples were stored at −30°C until use. The 21 neuroblastoma tissues were kindly provided by Dr. Wen-Ming Hsu (National Taiwan University Hospital, Taipei, Taiwan; IRB number 201407043RINC).

### DNA manipulation and plasmid construction

The *MYCN* gene was amplified from synthesized cDNA as described previously (Thermo Fisher Scientific). PCR was performed to generate pCMV6-XL4 plasmids (Invitrogen) with a full-length sequence of *MYCN*. The resulting plasmid (pCMV6-XL4-MYCN) was transformed into *Escherichia coli* (*E. coli*) strain DH5α and selected by ampicillin. The plasmid DNA (pCMV6-XL4-MYCN) was prepared and sequenced at the DNA Sequencing Facility (Genomics BioSci. & Tech., Taipei, Taiwan). In all, 2.5 × 10^5^ SK-N-AS cells were seeded 24 h before transfection in a 6-well plate using Lipofectamine 3000 (Invitrogen). Transiently transfected cells were harvested at 48 h post-transfection.

### RNA interference

The non-target siRNA or *MYCN*-siRNA (SMARTpool, Dharmacon, USA) were transfected into SK-N-DZ or SK-N-BE(2)-C cells to generate transient silencing of *MYCN* using Lipofectamine RNAiMAX (Invitrogen). In all, 4 × 10^5^ SK-N-DZ or SK-N-BE(2)-C cells were seeded on six-well plates 24 h before transfection, and harvested at 48 h post-transfection.

### qRT-PCR analysis

The cDNA sample was amplified and applied by using CFX Connect™ Real-Time PCR Detection System (Bio-Rad Laboratories). The mRNA expression values were measured by ΔΔCt and normalized to *GAPDH*. The qRT-PCR primer sequences were listed in Supplementary Table S1.

### Western blotting

Cells were lysed using lysis-C buffer [7 M urea, 2 M thiourea, 4% (w/v) CHAPS and 0.0002% (v/v) bromophenol blue] containing protease inhibitor (Bioman). The cells were homogenized on ice using an ultrasonic homogenizer (LABSONIC M ultrasonic homogenizer) with 60% amplitude and 0.6 cycle duration for 2 min. Cell lysate was centrifuged at 16,000 × *g* for 30 min at 4 °C. The supernatants were collected and measured protein concentrations with protein assay dye reagent (Bio-Rad Laboratories). Protein extracts were separated by SDS-PAGE and transferred onto a PVDF membrane (Millipore) and immunoblotted with antibodies. The membrane was blocked in 5% non-fat milk/PBST and incubated overnight with primary antibody diluted in blocking buffer at 4 °C: mouse anti-MYCN (abcam; 1:1000), rabbit anti-MTHFD2 (Genetex; 1:1000), rabbit anti-PAICS (Genetex; 1:1000), mouse anti-β-actin (Millipore; 1:5000), and mouse anti-α-tubulin (Genetex; 1:1000). The membrane was then treated with secondary HRP-conjugated antibody anti-rabbit or anti-mouse IgG (Sigma-Aldrich; 1:100,000) for 2 h at room temperature. Images were acquired using ECL substrate (BioRad) and FluorChem M (ProteinSimple).

### Luciferase reporter assay

Promoter regions of the *MTHFD2* and *PAICS* genes were amplified using PCR and cloned into the pGL4.18 vector (Promega) flanked with NheI and HindIII sites. The sequences of the promoter region primers are listed in Supplementary Table S2. SK-N-AS cells were seeded at 2.5 × 10^5^ per 6-well plate for 24 h. Then SK-N-AS cells were co-transfected with either 500 ng of *MTHFD2, PAICS* promoter luciferase reporters or pGL4.18 empty vector along with 10 ng of pGL4.74 Renilla luciferase plasmid DNA together with 500 ng of *MYCN* expression plasmid (pCMV6-XL4-MYCN) or control vector (pCMV6-XL4). At 5 h post-transfection, cells were recovered in completed DMEM for 1 h and then cells were maintained in completed DMEM containing 1 μl/ml 70% ethanol or 1 μg/ml tetracycline and incubated for 48 h. At 48 h post-transfection, cells were lysed with passive lysis buffer for 15 min at room temperature and the firefly and Renilla luciferase activities were measured with the Dual-Luciferase Reporter assay system (Promega) according to the manufacturer’s instructions.

### Generation of cell lines with stable knockdown of MTHFD2 and PAICS

SK-N-DZ cells were seeded at 4 × 10^5^ cells per 6-well plate for 24 h, and then transfected with 2 μg shRNA plasmid (RNAi core, IBMS, Academia Sinica, Taipei, Taiwan) which inhibited *MTHFD2* (shMTHFD2 #50 and #53), *PAICS* (shPAICS #74 and #75), *MTHFD2/PAICS* (shMTHFD2/PAICS) or *LacZ* (shRNA control) by lipofectamine 3000 (Invitrogen). Transfected cells were subsequently selected on 2 μg/ml puromycin to create the stable shRNA line. Stable cell subcultures were kept in DMEM medium containing 2 μg/ml puromycin (Supplementary Table S3).

### Cell harvest and extraction for targeted metabolomics assay

Cells were grown in 15-cm culture dish, during which the medium was replaced every day (DMEM supplemented with 10% fetal bovine serum and 2 μg/ml puromycin for stable clones) at 37 °C with 5% CO_2_ before extraction. Cells were collected at 80% confluence and rapidly rinsed with warm 0.9% NaCl isotonic saline three times before quenching. Then, 1 ml of ice cold water was added and flash frozen in liquid nitrogen and detached using a cell scraper. Cell suspension were lysed by freeze-thaw cycles twice, followed by sonication. Protein concentration were measured by BCA Protein Assay Kit for normalization. Metabolism was quenched and metabolites were extracted by 75:25 methanol:water extraction buffer. In brief, 900 μl of −20 °C methanol was add to 300 μl cell lysate and vigorously vortexed and then centrifuged at 14,000 × *g* for 20 min at 4 °C. The supernatants were collected and dried using a centrifugal evaporator and stored at −80 °C prior to analysis^29^.

### Instrumentation, method development, and data analysis for targeted metabolism analysis

The complete LC-MS platform consists of HPLC system (Agilent 1260 series, Germany) equipped with a time-of-flight mass spectrometer (micrOTOF-QII, Bruker Daltonik, Bremen, Germany), controlled by Bruker Daltonics Hystar 3.2 software. Liquid chromatography separation was achieved on a hypersphere C18 column (250 mm × 4.6 mm, 5 μm particle size, YMC, America), using reversed phase chromatography with the ion pairing agent tributylamine in the aqueous mobile phase to enhance retention and separation. The total run time was 30 min and the injection volume was 20 μl. Every dried sample was dissolved in 400 μl solution which contained H_2_O:ACN:MeOH at a ratio of 1:1:2 with 1% formic acid (FA). The flow rate was 500 μl/min. Solvent A was 3% methanol with 10 mM tributylamine and 15 mM acetic acid; solvent B was 100% acetonitrile/0.1% FA. The gradient was 0 min, 0% B; 2.5 min, 0% B; 5 min, 20% B; 7.5 min, 20% B; 13 min, 55% B; 20 min, 90% B; 25 min, 90% B; 25.1 min, 0% B; and 30 min, 0% B.

An electrospray ionization interface was used to direct column eluent to the mass spectrometer. Mass spectra in the range m/z 50–500 were obtained by electrospray ionization in negative-ion mode. Initial instrument optimization was performed by infusing a mixture of L-serine (m/z 104.0288; Cat. No.: SI-S4500, Sigma-Aldrich), AICAR (m/z 257.0790; Cat. No.: SI-A9978, Sigma-Aldrich), IMP (m/z 347.0398; Cat. No.: SI-I4625, Sigma-Aldrich), and GMP (m/z 362.0507; Cat. No.: SI-G8377, Sigma-Aldrich), using a syringe pump (KdScientific, Holliston, MA). Various instrumental settings were optimized to maximize the signal with the final parameters as follow: gas temperature 200 °C, drying gas flow rate 8.0 L/min, nebulizer gas pressure 4.0 bar, and capillary and endplate offset potentials were 2500 V and 500 V, respectively.

### Cell cycle analysis

Stable shRNA expression cells were collected, fixed in 70% ethanol, and stored at −20 °C overnight. The cells were washed with PBS and resuspended in PBS containing 100 μg/ml RNase A and 0.1% Triton X-100, then incubated at 37 °C for 1 h. Cells were stained with 5 μg/ml propidium iodide (Santa Cruz). The DNA content of the cells was analyzed using a FACSCanto instrument (BD Biosciences Immunocytometry Systems). Ten thousand cells were collected for each measurement. The percentage of cells in different phases of the cell cycle was analyzed using ModFit LT (Verity Software House).

### Cell proliferation, colony formation, and migration assays

For MTS analysis, stable shRNA expression cells (shLacZ, shMTHFD2#50, shMTHFD2#53, shPAICS#74, shPAICS#75, and shMTHFD2/PAICS) were seeded in 96-well plate at 4000 cells per well with DMEM containing 2 μg/ml puromycin. At each 24 h, 48 h, 72 h time point, 20 μl of MTS/PMS (Promega) were added and incubated for 2 h at 37 °C with 5% CO_2_. The cell viability was determined by OD 490 nm using an ELISA reader (BioRad). For colony formation assay, the stable shRNA expression cells were seeded in 6-well plates (600 cells/well) with DMEM containing 2 μg/ml puromycin and incubated for 2 weeks. After 2 weeks, the colonies were fixed with methanol overnight and then stained by 4% GIEMSA stain for 1 h and analyzed by ImageJ. For cell migration, 3 × 10^4^ stable shRNA expression cells with 1% FBS medium were loaded into the inserts, and medium containing 10% FBS was loaded into the lower compartments of an 8-μM pore size Transwell plate (Corning). The cells were incubated at 37 °C with 5% CO_2_ for 6 h, then fixed for 30 min with 100% methanol and stained with 1% GIEMSA for 30 min. Cotton swabs were used to remove cells from the upper side of the inserts. Images of five different microscope fields of each insert were captured and counted.

### Identification of potential compounds suppressing *MTHFD2* or *PAICS* expression

Perturbation datasets corresponding to the treatment of various drugs or compounds for ten cell lines, including A375, A549, HA1E, HCC515, HEPG2, HT29, MCF7, NPC, PC3, and VCAP, were obtained from the Library of Integrated Network-based Cellular Signatures (LINCS) project^30^. To identify the compounds that significantly suppress the gene expressions of *MTHFD2* and *PAICS* based on the perturbation datasets, computational approach was performed as detailed in our previous methods^31,32^. Briefly, the expression changes of all measured transcripts (*n* = 12,494) were ordered from the most downregulated (negative) to the most upregulated (positive) cross ten cell lines and assessed if the gene of interest was significantly enriched on the down-regulation edge cross all cell types under the perturbation of the given compound. Finally, the expressions of *MTHDF2* and *PAICS* were found to be suppressed by anisomycin and apicidin, respectively.

### Drug combination assay

SK-N-DZ, SK-N-BE(2)-C, SK-N-AS, or SK-N-SH neuroblastoma cells were seeded at 5000 cells per well in 96-well plate for 24 h before drug treatments. To prepare the stock solution, anisomycin (CAS No.: 22862-76-6; Selleckchem) and apicidin (CAS No.: 183506-66-3; ApexBio) were dissolved in dimethyl sulfoxide (DMSO, Sigma-Aldrich) to have a final concentration of 0.1, 0.2, and 0.4 µM respectively. To test the synergistic effect of anisomycin and apicidin combination, the corresponding combination solutions were prepared proportionally at 1:1 molar ratio. The control groups received an equivalent amount of DMSO as corresponding treatment groups. At the end of 24 and 48 h of treatments, 20 μl of MTS/PMS (Promega) were added and incubated for 2 h at 37 °C with 5% CO_2_. The cell viability was determined by OD 490 nm using an ELISA reader (BioRad) and normalized with the corresponding control groups. Drug synergy was evaluated using CompuSyn (http://www.combosyn.com) to calculate the combination index (CI) values: synergism (CI < 0.9), additive effect (CI = 0.9–1.1), and antagonism (CI > 1.1)^33^.

### Cell apoptosis assessment

In all, 1 × 10^6^ SK-N-DZ or SK-N-BE(2)-C cells were seeded in a 10-cm culture dish for 24 h before drug treatments. For apoptosis detection, 0.2 µM anisomycin, 0.2 µM apicidin, and the combination of solutions were prepared proportionally at 1:1 molar ratio. At the end of 24 h of treatment, cells underwent PBS washes before treated with the FITC Annexin V Apoptosis Detection Kit I (BD Pharmingen, USA) according to the manufacturer’s instructions. Ten thousand stained cells were acquired on a FACSCanto instrument (BD Biosciences Immunocytometry Systems) for each measurement.

### Statistical analysis

Results were expressed as the mean ± standard deviation (SD). The Student’s *t*-test was used to analyze differences between two groups. The differences between groups were considered to be statistically significant when *P* < 0.05. All experiments were performed in triplicate.

## Results

### Integrated analysis of multi-layer omics data reveals higher cellular metabolism in MNA neuroblastoma

**Fig. 1:**
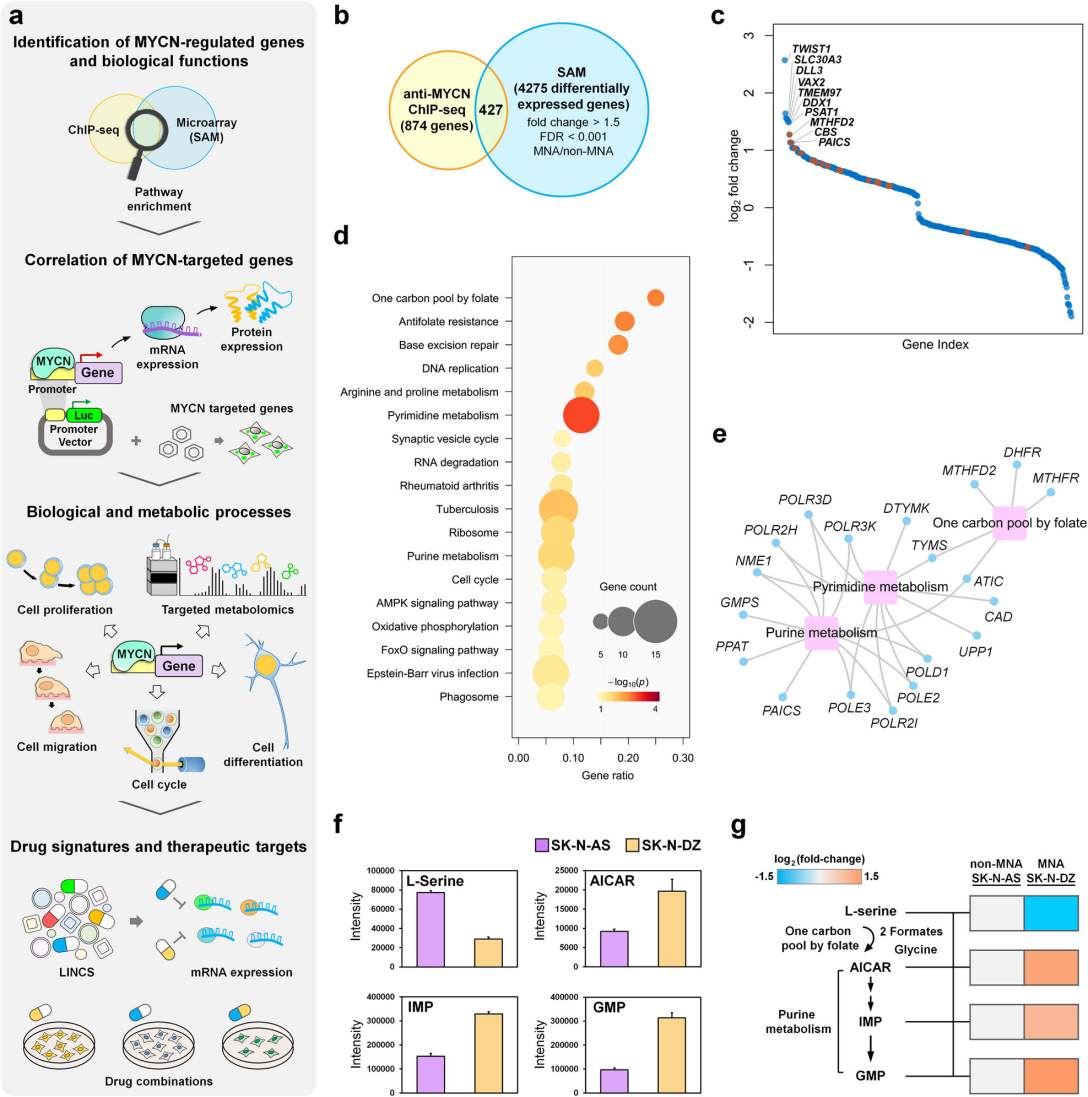
Integrative analysis of anti-MYCN ChIP-seq and gene expression profile of neuroblastoma patients. **a** Schematic representation of the experimental design. **b** Venn diagram showing the intersection of MYCN-bound genes of anti-MYCN ChIP-seq and differentially expressed genes (between HR-MNA and HR-non-MNA samples). **c** Scatter plot revealing fold-change expression rank of the 427 MYCN-regulated genes. Metabolic genes that illustrate in gene-pathway association networks (**e**) are highlighted in red. **d** The KEGG pathway enrichment of the 427 MYCN-regulated genes. The color and size of the node represent the *p*-value and the number of genes mapped to the indicated pathways, respectively. **e** The MYCN-regulated gene-pathway association networks, where the blue nodes and magenta rectangles correspond to genes and KEGG pathways, respectively. The gray lines represent the genes participating in the indicated pathways. **f** Targeted metabolomics assay was performed to measure the relative levels of L-serine, nucleoside intermediate (AICAR), and nucleoside monophosphates (IMP and GMP) between MNA SK-N-DZ and non-MNA SK-N-AS neuroblastoma cell lines; bar plot showing the intensity of each metabolite measured in technical replicates. **g** Relative abundance of metabolite levels in SK-N-DZ were normalized to SK-N-AS cells. The Log_2_ fold-change of L-serine, AICAR, IMP, and GMP are −1.47, 1.31, 1.00, and 1.49, respectively. At least two technical repeats for each of two biological replicates were performed

To identify highly confident MYCN-regulated genes, we combined genes that differentially expressed between *MYCN* status with MYCN-bound genes (Fig. 1a). Here, we compiled a neuroblastoma gene expression cohort from six independent studies and identified 4275 differentially expressed genes between HR-MNA and HR-non-MNA samples. By integrating with 874 MYCN-bound genes that identified in our previous anti-MYCN ChIP-seq experiment^27^, of which 427 common genes were found to be regulated by MYCN (Fig. 1b and Supplementary Table S4), and we termed these genes as potential MYCN-regulated genes. A few of these genes have been verified as direct transcriptional targets of MYCN, such as *TWIST1*, *DLL3*, and *DDX1* (Fig. 1c)^34–36^. The functional enrichment analysis of the potential MYCN-regulated genes revealed cancer hallmark-associated biological processes such as cell cycle and metabolic pathways (Fig. 1d and Supplementary Table S5).

Previous studies have revealed that amplification of *MYCN* increases neuroblastoma cell growth through glutathione biosynthesis and glutamine uptake, thus suggesting MYCN reprograms neuroblastoma metabolism^11,37,38^. In our analysis, one carbon pool by folate, pyrimidine metabolism and purine metabolism were significantly altered by MYCN (Fig. 1e). One carbon pool by folate metabolism regulates the conversion of serine to glycine in mitochondria and releases the production of folate to cytosol for purine synthesis. Interestingly, a co-localization of purinosome and mitochondria was also reported under purine-depleted condition^25^. To investigate whether MNA neuroblastoma favors one carbon metabolism and purine metabolism, we first assessed the intrinsic differences of MNA and non-MNA neuroblastoma cells for serine consumption and nucleoside production. Targeted metabolic assay was performed to quantify the relative levels of serine, nucleoside intermediate (AICAR), and nucleoside monophosphate (IMP and GMP) between MNA SK-N-DZ and non-MNA SK-N-AS cell lines (Fig. 1f and Supplementary Fig. 1). The levels of AICAR, IMP, and GMP were significantly higher in MNA neuroblastoma compared to non-MNA neuroblastoma cells, suggesting MNA neuroblastoma may require higher demand in purine biosynthesis for rapid cell proliferation (Fig. 1g). In addition, we observed a lower level of serine in MNA neuroblastoma cells which could be converted into glycine in one carbon pool by folate pathway (Fig. 1g). Collectively, these data suggest that MYCN may play a role in regulating metabolism in neuroblastoma through potential MYCN-regulated genes.

### *MTHFD2* and *PAICS* expressions are strongly correlated to *MYCN* status in neuroblastoma

**Fig. 2:**
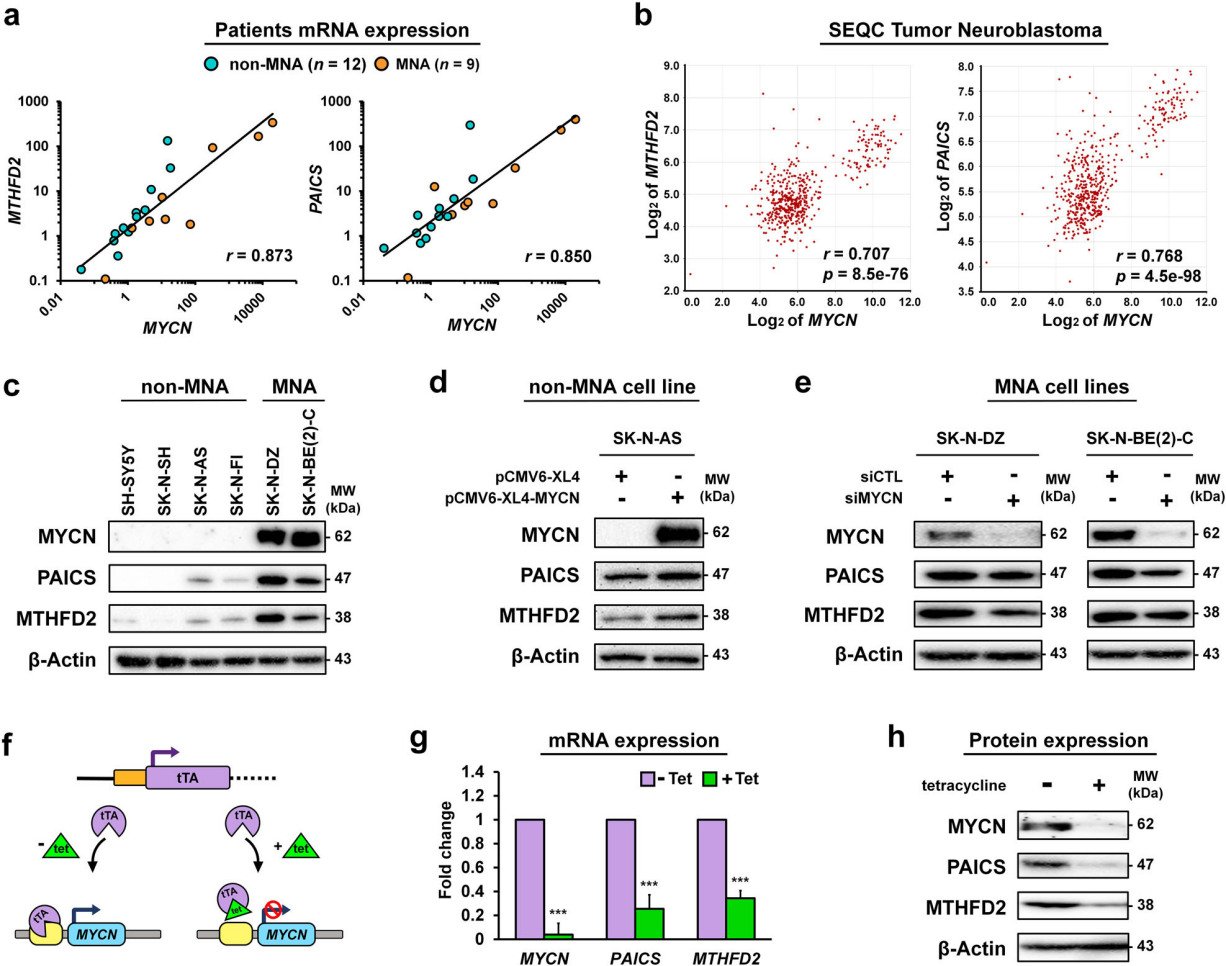
Positive correlation of *MTHFD2* and *PAICS* with *MYCN* in neuroblastoma. **a** The *MYCN, MTHFD2*, and *PAICS* mRNA expression levels of MNA (*n* = 9) and non-MNA (*n* = 12) tissue samples from neuroblastoma patients were assessed by qRT-PCR analysis. *MTHFD2* and *PAICS* were positively correlated to *MYCN* amplification with *r* = 0.873 and 0.850, respectively. **b** Correlation of *MTHFD2* and *PAICS* to *MYCN* mRNA levels was assessed using the SEQC-NB498 dataset from R2 platform (http://r2.amc.nl/). **c** The results from western blot analyses demonstrating a positive correlation of MTHFD2 and PAICS with MYCN expression in six neuroblastoma cell lines. **d** Non-MNA SK-N-AS cells were transfected with pCMV6-XL4-MYCN, both PAICS and MTHFD2 were elevated along with MYCN. **e** MNA SK-N-DZ and SK-N-BE(2)-C cells were transfected with small interfering RNA (siRNA) to silence MYCN. Protein expression of MYCN was effectively suppressed where PAICS and MTHFD2 were concomitantly downregulated. **f** The Tet-21N cells express MYCN in the absence of tetracycline, where the addition of tetracycline inhibits tTA to bind to the promoter of MYCN thus gene expression is turned off. **g**, **h** After 48 h of tetracycline treatment, both MTHFD2 and PAICS were significantly downregulated following MYCN depletion at mRNA (**g**) and protein levels (**h**). The relative mRNA expressions were calculated by qRT-PCR analysis, and normalized to *GAPDH* gene. ***P < 0.001

We speculate that high levels of cellular metabolism are linked to neuroblastoma progression, thus two highly expressed metabolic genes *MTHFD2* and *PAICS* among one carbon pool by folate and purine metabolism pathways in MNA neuroblastoma prompted us to further examine their relationship with *MYCN* status (Fig. 1a, c, e). We first evaluated the mRNA expression levels of *MTHFD2, PAICS*, and *MYCN* in 21 tissue samples from neuroblastoma patients (MNA: *n* = 9; non-MNA: *n* = 12). The mRNA expressions of *MTHFD2* and *PAICS* were positively correlated to *MYCN* by qRT-PCR with a Pearson correlation coefficient (*r)* of 0.873 and 0.850 respectively (Fig. 2a). In consistent with our finding, the SEQC cohort results showed that mRNA expressions of *MTHFD2* and *PAISC* were positively correlated with *MYCN*, and highly expressed in MNA patients (Fig. 2b and Supplementary Fig. 2). Next, we assessed the protein expression levels in six neuroblastoma cell lines, where the MNA cell lines had markedly higher endogenous expressions of MTHFD2 and PAICS compared to non-MNA cell lines (Fig. 2c and Supplementary Fig. 3).

To further confirm the regulation of MYCN with MTHFD2 and PAICS, we assessed the expression response upon MYCN perturbation in MNA and non-MNA cell lines. Overexpression of MYCN was introduced by pCMV6-XL4-MYCN in non-MNA SK-N-AS cells, both PAICS and MTHFD2 were elevated along with MYCN (Fig. 2d). Contrastingly, silencing MYCN in MNA SK-N-DZ and SK-N-BE(2)-C cells using small interfering RNA (siRNA) revealed that PAICS and MTHFD2 expressions were concomitantly downregulated with MYCN (Fig. 2e). Moreover, the tetracycline inducible system was also applied to evaluate the effects on neuroblastoma. The Tet-21N cells express *MYCN* in the absence of tetracycline, whereas the addition of tetracycline inhibits the tTA to bind to the promoter of MYCN thus gene expression is turned off (Fig. 2f). After 48 h of tetracycline treatment, both MTHFD2 and PAICS were significantly downregulated following MYCN depletion at mRNA and protein levels indicating that MYCN might influence the transcription efficiency of MTHFD2 and PAICS (Fig. 2g, h).

### MYCN transcriptionally targets MTHFD2 and PAICS in neuroblastoma

**Fig. 3:**
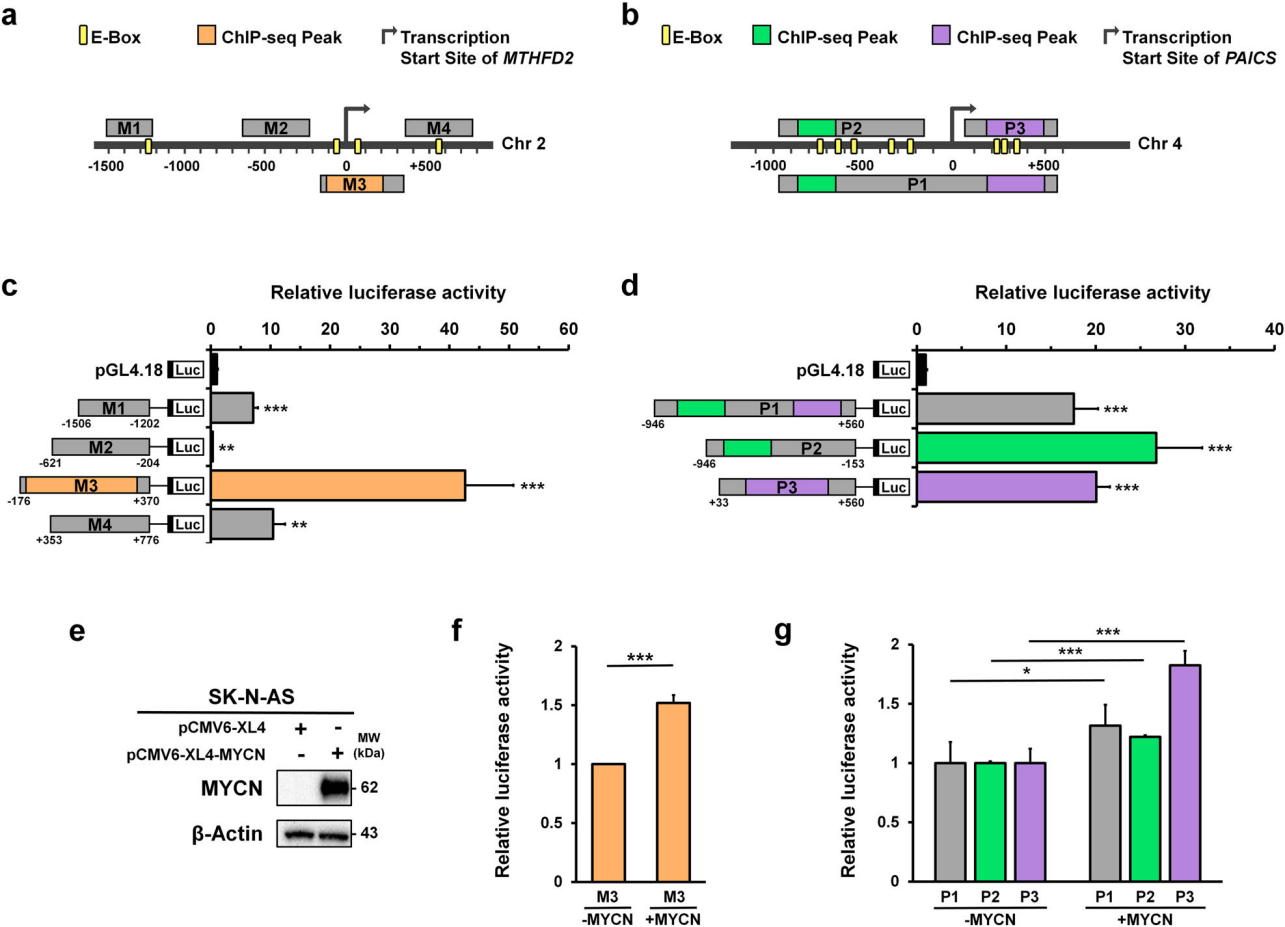
*MTHFD2* and *PAICS* are the MYCN target genes. **a** Schematic representation of the four *MTHFD2* DNA fragments including M1: the most upstream of transcription start site (TSS) containing E-box; M2: the upstream of TSS without E-box; M3: located across the up- and down- stream of TSS and overlapped with MACS_peak_36001 containing E-boxes; and M4: the downstream of TSS with a E-box. **b** Schematic representation of the three *PAICS* DNA fragments including P1: located across the up- and down- stream of TSS which contained both MACS_peak_50055 and MACS_peak_50056; P2: upstream of TSS with MACS_peak_50055; and P3: downstream of TSS with MACS_peak_50056. **c**, **d** Luciferase reporter assay was performed to measure the relative luciferase activity of *MTHFD2* (**c**) and *PAICS* (**d**) constructs. **e**–**g** Non-MNA SK-N-AS cells were transfected with pCMV6-XL4 or pCMV6-XL4-MYCN. **e** The western blot analyses demonstrating the efficiency of transfection. **f**, **g** *MYCN* overexpressed SK-N-AS and SK-N-AS control cells that transfected with *MTHFD2* and *PAICS* constructs indicating the binding sequences of *MTHFD2* (**f**) and *PAICS* (**g**) are significantly upregulated by *MYCN* expression. *P < 0.05; **P < 0.01; ***P < 0.001

Our previous anti-MYCN ChIP-seq data revealed that *MTHFD2* and *PAICS* had significant MYCN binding signals comparing with IgG control (Supplementary Fig. 4). To examine whether MYCN directly drives *MTHFD2* or *PAICS*, we applied a dual luciferase reporter assay to determine the MYCN binding sites on *MTHFD2* and *PAICS* promoters (Fig. 1a). According to the ChIP-seq results and examination of consensus E-box motifs, four *MTHFD2* (M1, M2, M3, and M4) and three *PAICS* (P1, P2, and P3) promoter sequences were constructed using the pGL4.18 luciferase vector and co-transfected with pCMV6-XL4-MYCN in MNA SK-N-AS cell lines (Fig. 3a, b). The luciferase activity of M1, M3, and M4 were significantly enhanced, of which M3 that consisted the MYCN ChIP binding peaks resulted the highest activity with a 42‑fold change relative to the empty pGL4.18 vector (Fig. 3c). On the other hand, P1, P2, and P3 constructs containing the ChIP-seq binding peaks were increased by the presence of MYCN vector compared to empty pGL4.18 (Fig. 3d). Moreover, we compared the MYCN overexpressed SK-N-AS with SK-N-AS control (Fig. 3e), and also observed that the binding sequences of *MTHFD2* and *PAICS* constructs were significantly upregulated by *MYCN* expression (Fig. 3f, g).

### MTHFD2 and PAICS regulate purine biosynthesis in MNA neuroblastoma

**Fig. 4:**
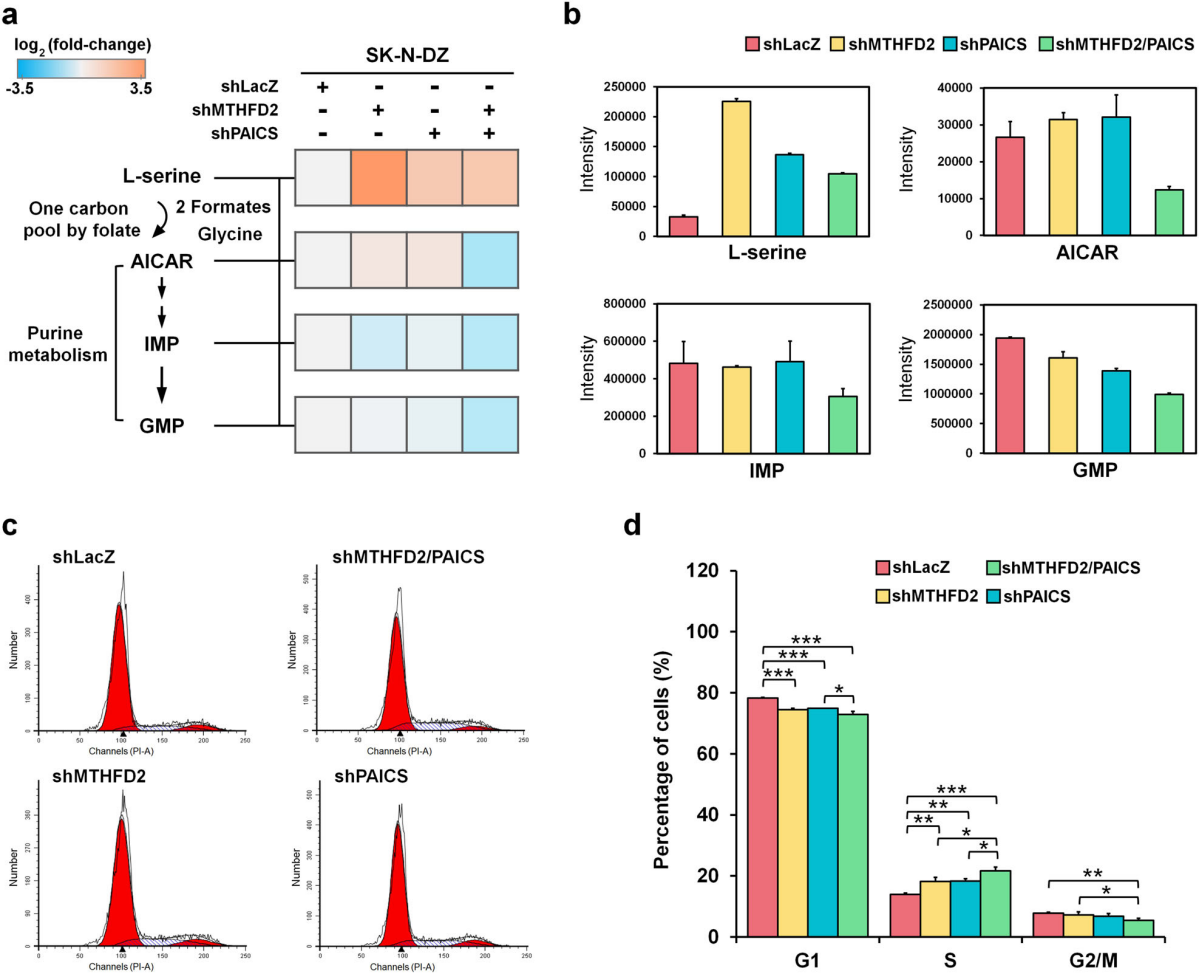
Dual knockdown of MTHFD2 and PAICS depletes purine nucleoside production and interrupts cell cycle in MNA neuroblastoma. **a** Metabolites levels of L-serine, AICAR, IMP and GMP were normalized to shLacZ SK-N-DZ cells. The log_2_ fold-change of L-serine in knockdown cells (shMTHFD2: 3.76; shPAICS: 1.65; and shMTHFD2/PAICS: 1.68), the log_2_ fold-change of AICAR in knockdown cells (shMTHFD2: 0.47; shPAICS: 0.52; and shMTHFD2/PAICS: −1.03), the log_2_ fold-change of IMP in knockdown cells (shMTHFD2: −0.48; shPAICS: −0.16; and shMTHFD2/PAICS: −0.87), and the log_2_ fold-change of GMP in knockdown cells (shMTHFD2: −0.02; shPAICS: −0.15; and shMTHFD2/PAICS: −0.83). At least two biological replicates for each metabolite were performed. **b** The intensity of L-serine, AICAR, IMP, and GMP in shLacZ, shMTHFD2, shPAICS, and shMTHFD2/PAICS SK-N-DZ cells. **c** The distribution of cells in the different phases of the cell cycle was analyzed by flow cytometry. **d** The percentage of cells in the S phase of shMTHFD2 and shPAICS cells increased, by 4.29% and 4.34%, respectively, and that of dual knockdown cells dramatically increased by 7.70% relative to shLacZ control of SK-N-DZ cells. The percentage of cells decreased in both G1 and G2/M phases, along with increased cell population of S phase, were observed in shMTHFD2, shPAICS, and dual knockdown. Of note, dual knockdown decreased by 5.33% and 2.36% of cell population in the G1 and G2/M phases, respectively. *P < 0.05; **P < 0.01; ***P < 0.001

To explore the impact of the two metabolic enzymes on MNA neuroblastoma, we first constructed stable knockdown of LacZ (control), MTHFD2, PAICS, and MTHFD2/PAICS (dual-knockdown) by delivering shRNA hairpins into MNA SK-N-DZ cells followed by puromycin selection (Supplementary Fig. 5). In one carbon pool by folate pathway, serine is catabolized in mitochondria to produce formaldehyde, and then transforms into essential molecule, 10-formyl-THF, for purine biosynthesis by MTHFD2^39^. To investigate the relationship of one carbon pool by folate and purine biosynthesis in neuroblastoma, we analyzed the relative abundance of metabolites using liquid chromatography mass spectrometry (Fig. 1a and Supplementary Fig. 6). We found shMTHFD2, shPAICS, and shMTHFD2/PAICS SK-N-DZ cells decreased the consumption of serine, and this observation implies that knockdown of either MTHFD2 or PAICS suppresses the metabolic activity in one carbon pool by folate pathway (Fig. 4a, b).

On the other hand, 5-aminoimidazole-4-carboxamide ribonucleotide (AICAR), the final intermediate for assembling inosine monophosphate (IMP), and guanosine monophosphate (GMP)^40^ were markedly reduced in shMTHFD2/PAICS relative to single knockdown. Thus, we suggest that the depletion of AICAR level limits the production of IMP and GMP when both MTHFD2 and PAICS are suppressed (Fig. 4a, b). Of note, the single knockdown of either MTHFD2 or PAICS elevated the levels of AICAR compared to the control. The results suggest that the synergistic effect of MTHFD2 and PAICS are related to the biosynthesis of nucleotides, as well as their interconnection in one carbon by folate and purine metabolism in MNA neuroblastoma cells.

Since the targeted metabolomics analysis indicated an imbalance of purine nucleotides in dual knockdown cells, we hypothesized that the cell cycle perturbations might be induced. Flow cytometry for DNA content was conducted and the distribution of cells in different phases of the cell cycle was analyzed. The results showed that the percentage of cells in the S phase of shMTHFD2 and shPAICS cells increased, by 4.29% and 4.34%, respectively, and that of dual knockdown cells increased by 7.70% relative to shLacZ control of MNA SK-N-DZ cells, indicating an interruption of the transition from S to G2/M phases (Fig. 4c, d). The percentage of cells decreased in both G1 and G2/M phases, along with increased cell population of S phase, especially the dual knockdown cells which decreased by 5.33% and 2.36% of population in the G1 and G2/M phases, respectively (Fig. 4c, d). Thus, purine intermediates depletion and S phase arrest may be correlated with dysregulation of DNA synthesis.

### Dual knockdown of MTHFD2 and PAICS suppresses neuroblastoma cell growth

**Fig. 5:**
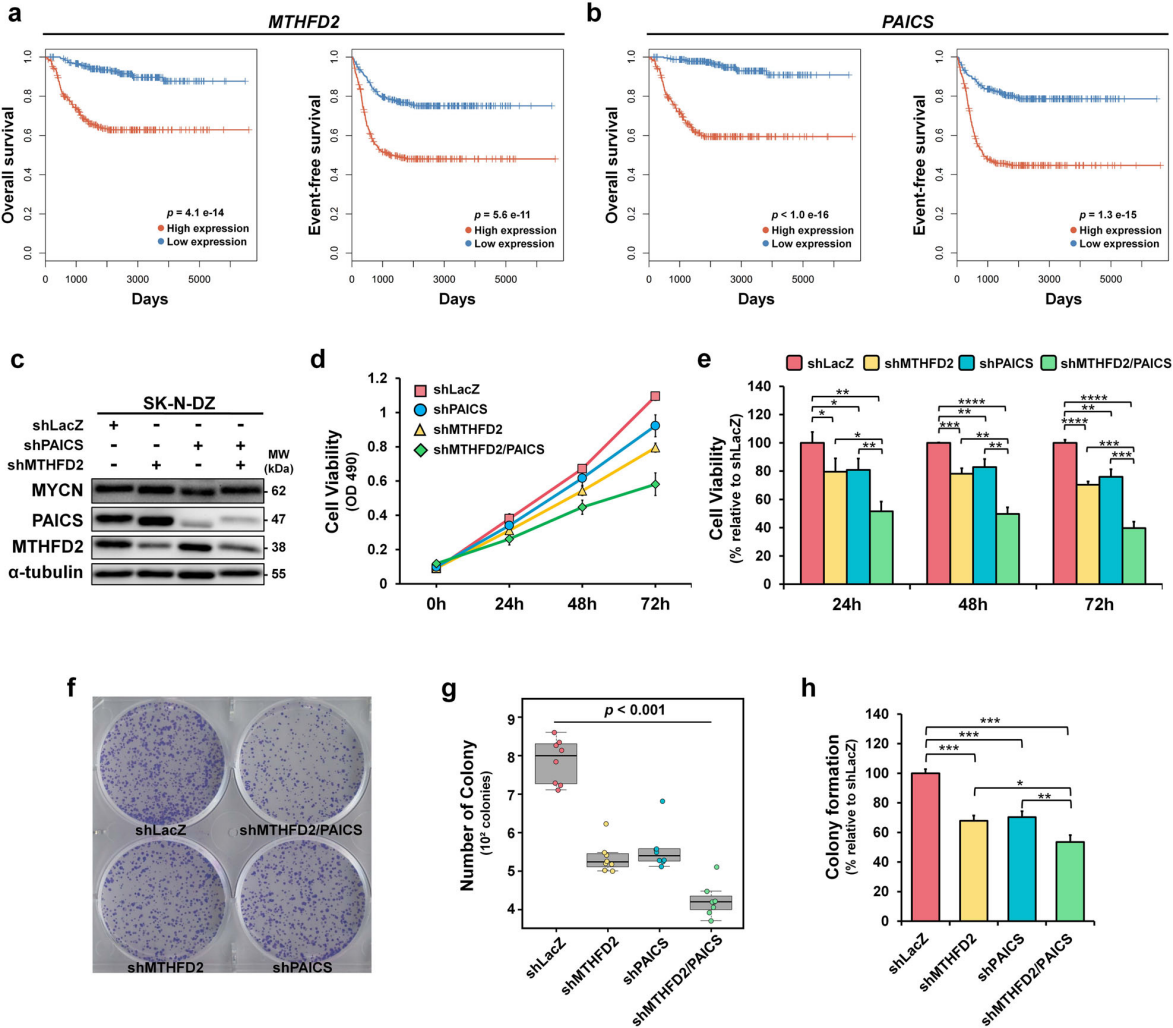
MTHFD2 and PAICS are related to neuroblastoma cell growth. **a**, **b** Kaplan–Meier curves showing significant poorer prognosis in overall and event-free survival for patients with high expressions of *MTHFD2* (**a**) and *PAICS* (**b**) using SEQC NB-498 dataset. The samples were straitified into high and low expressions based on the median value of the given gene expression. The differences in survival time between two groups were assessed by the log-rank test. **c** Western blot analyses revealing the SK-N-DZ stable knockdown cell lines of shRNA control, MTHFD2, PAICS, and MTHFD2/PAICS. **d** Cell viability of the SK-N-DZ stable knockdown cell lines was measured by MTS assays. **e** The cell viability in dual knockdown of MTHFD2 and PAICS (shMTHFD2/PAICS) has declined 48.4%, 50.2%, and 60.3% at 24, 48, and 72 h respectively. **f**–**h** The effects of SK-N-DZ stable knockdown cell lines on colony formation ability in representative plates (**f**) and quantitative analysis (**g**). **h** Single knockdown of MTHFD2 or PAICS alone was associated with approximately 32.1% and 29.6% declension, respectively, whereas dual knockdown of both MTHFD2 and PAICS resulted in a decrease of colony number to 46.5% compared with shLacZ cells

To elucidate the prognostic implications of MTHFD2 and PAICS in neuroblastoma progression, we analyzed the association of *MTHFD2* and *PAICS* expressions with patient survival. Poor overall and event-free survivals were found in highly expressed *MTHFD2* or *PAICS* neuroblastoma patients (Fig. 5a, b). According to the impact of *MTHFD2* and *PACIS* on neuroblastoma patient survival, we ascertain their stimulatory effects on cell proliferation by cell viability and colony formation assays in response to MTHFD2 and PAICS expression manipulation (Figs. 1a, 5c–h and Supplementary Fig. 7). Cell viability was measured at 24, 48, and 72 h by MTS assays, and the results indicated a noticeable decline in cell proliferation in response to MTHFD2 and PAICS. Cell proliferation was strongly affected by MTHFD2 and PAICS; the viability of shMTHFD2 cells declined by 20. 4%, 20.8%, and 29.7%, at 24, 48, and 72 h, and the viability of shPAICS cells also decreased by 19.2%, 17.2%, and 24.0% at 24, 48, and 72 h respectively (Fig. 5d, e). Either shRNA-mediated knockdown of MTHFD2 or PAICS reduced cell viability compared with shLacZ control (Supplementary Fig. 8). However, the most significant reduction in cell viability occurred in dual knockdown of MTHFD2 and PAICS (shMTHFD2/PAICS) where the cell viability declined 48.4%, 50.2%, and 60.3% at 24, 48, and 72 h respectively (Fig. 5d, e).

Colony formation was monitored to investigate the long-term effects of MTHFD2 and PAICS on neuroblastoma cell proliferation (Fig. 5f–h). Single knockdown of MTHFD2 or PAICS alone was associated with ~32.1% and 29.6% declension of colony formation ability respectively, whereas dual knockdown of both MTHFD2 and PAICS resulted in a decrease of colony number to 46.5% compared with shLacZ cells (Fig. 5h). These observations suggest that simultaneous knockdown of MTHFD2 and PAICS further impair the MNA cell proliferation, and they may serve as a regulatory purpose in cell growth together.

### Dual knockdown of MTHFD2 and PAICS suppresses neuroblastoma cell migration

**Fig. 6:**
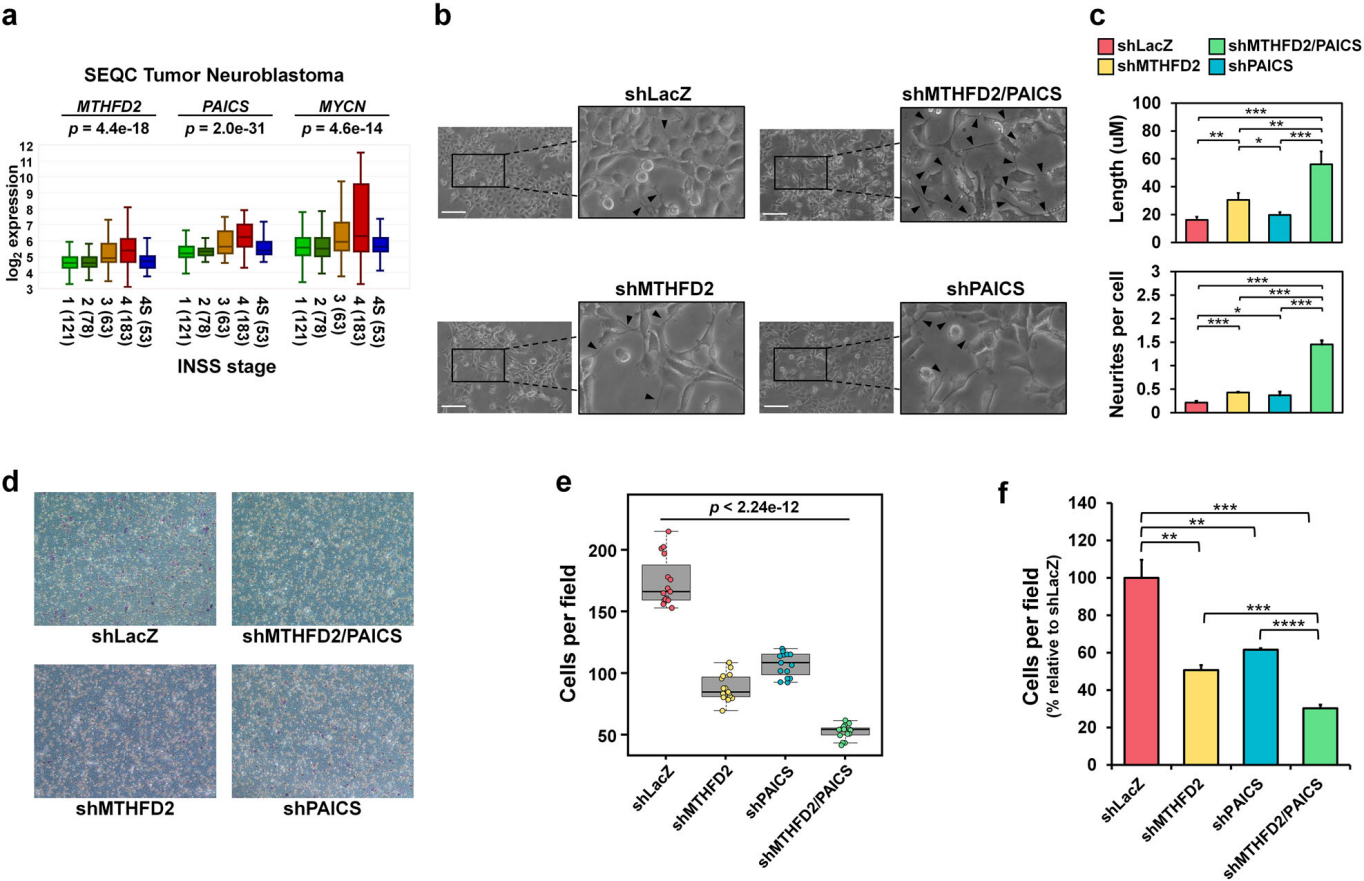
MTHFD2 and PAICS are correlated with metastatic tumor stage, neurite outgrowth, and cell migration in neuroblastoma. **a** Expressions of *MTHFD2*, *PAICS*, and *MYCN* correlate with metastatic stage (4) as compared with localized stages (1–3) in neuroblastoma patients (http://r2.amc.nl/; Tumor Neuroblastoma –SEQZ – 498 – RPM – seqcnb1). The *p*-values were derived from ANOVA. **b** Phase contract microscopic images of stable single and dual knockdown of MTHFD2 and PAICS in MNA SK-N-DZ cells. A neuronlike cell morphology was observed in dual knockdown shMTHFD2/PAICS SK-N-DZ cells in comparison with single knockdown or control cells. Black arrow: outgrowth of neurites; Scale bar: 100 μm. **c** The average neurite length and number of neurites per cell were significantly increased in shMTHFD2/PACIS cells. **d**, **e** The images (**d**) and the quantitative analysis (**e**) showing the number of migrated cells in stable shLacZ, shMTHFD2, shPAICS, and shPAICS/MTHFD2 SK-N-DZ cells. **f** shMTHFD2/PAICS cells was dramatically decreased by 69.7% relative to shLacZ control. Single knockdown of MTHFD2 decreased by 49.3%, while that of PAICS decreased by 38.4% relative to shLacZ control

To further investigate the impacts of MTHFD2 and PAICS on tumor aggressiveness, we analyzed the relationship of *MTHFD2* and *PAICS* expressions with tumor stages (INSS). A higher *MTHFD2* or *PAICS* expression was found in stage 4 compared with stages 1–3 and 4S, which had a similar expression pattern as *MYCN* in INSS stages (Fig. 6a). The above results suggest that *MTHFD2* and *PAICS* are correlated with metastatic stage and tumor progression. We also observed a differentiated cell morphology in dual knockdown shMTHFD2/PAICS cells in comparison with single knockdown or shLacZ control cells which is linked to a less aggressive neuronlike phenotype in neuroblastoma (Fig. 6b)^4,11^. The shLacZ control cells exhibiting typical rounder morphology compared with single or dual knockdown MNA neuroblastoma cells, whereas the dual knockdown shMTHFD2/PAICS cells exhibited a noticeable outgrowth of neurite lengths and numbers of branch points (Fig. 6b, c). These morphological changes, together with the correlation of *MTHFD2* or *PAICS* expression to INSS, led us to probe whether MTHFD2 and PAICS play roles in malignant transformation and tumor aggressiveness.

Cell migration is a crucial process during metastasis, thus we performed transwell assay to determine the migration ability on MTHFD2 and PAICS (Fig. 6d–f). Dual knockdown of MTHFD2 and PAICS had a significantly poorer migration ability compared with single knockdown of MTHFD2 and PAICS alone where shMTHFD2/PAICS was dramatically decreased by 69.7% compared to shLacZ control (Fig. 6f). As in the single knockdown results, shMTHFD2 cells decreased by 49.3%, while that of shPAICS cells decreased by 38.4% compared to shLacZ control, suggesting silencing of MTHFD2 and PAICS had synergistic effect on migration ability in MNA neuroblastoma (Fig. 6f).

### Compound perturbagens suppressing *MTHFD2* and *PAICS* show synergistic effects on MNA neuroblastoma

**Fig. 7:**
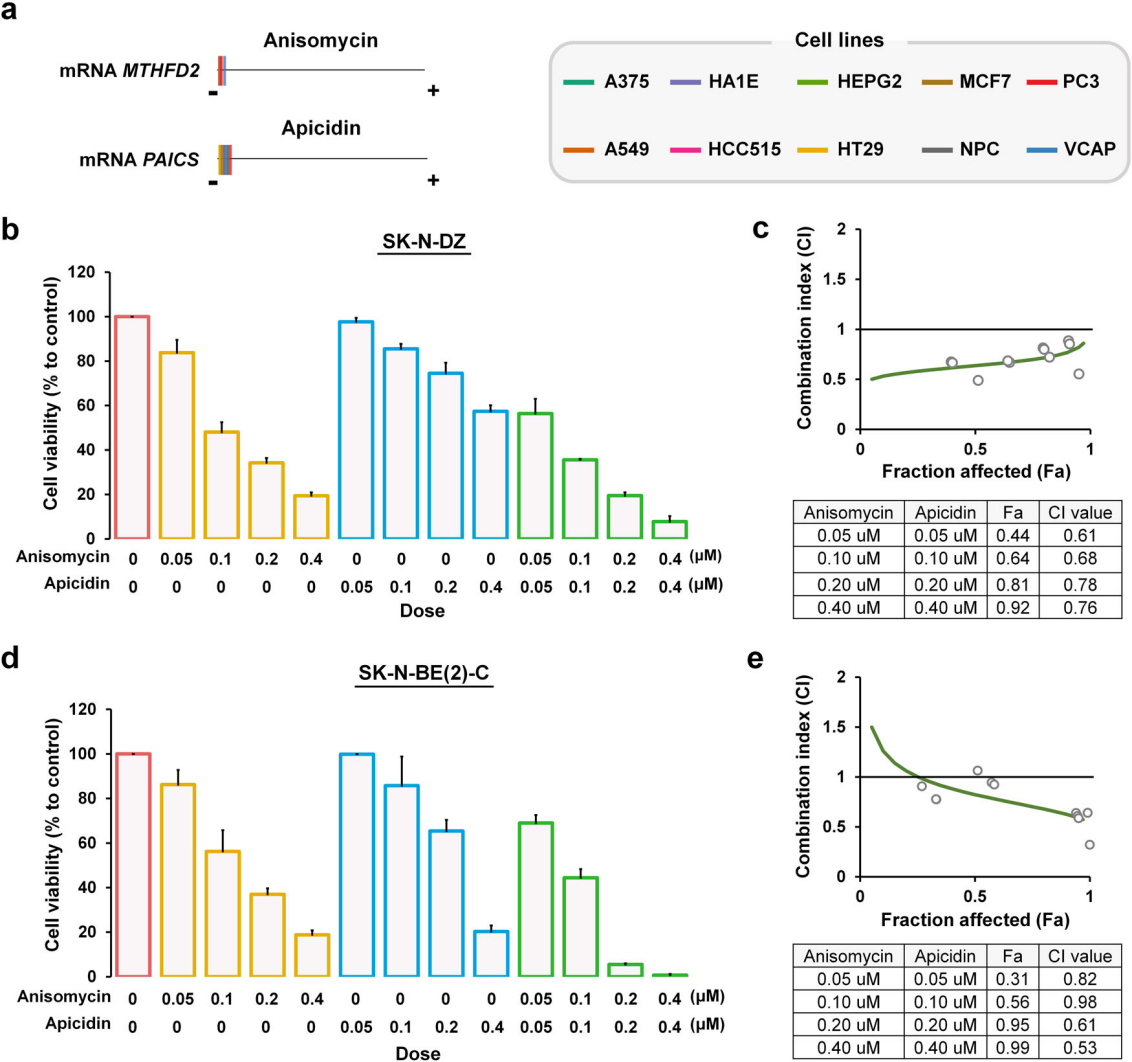
Combinatorial targeting of *MTHFD2* and *PAICS* inhibits MNA neuroblastoma cell proliferation. **a** The relative transcriptional changes of *MTHFD2* and *PAICS* after anisomycin or apicidin treatment for 24 h across ten cell lines. For each cell type, the expression changes of measured transcripts (*n* = 12,494) were ordered from the most downregulated (the negative side) to the most upregulated gene (the positive side), from which *MTHFD2* (upper left panel) or *PAICS* (lower left panel) was indicated by a colored line. **b**–**e** The combined effects of anisomycin and apicidin on MNA neuroblastoma cell lines. Percent cell growth of MNA SK-N-DZ (**b**) and SK-N-BE(2)-C (**d**) cells relative to corresponding controls treated with anisomycin, apicidin, or the combined treatment at indicated dosage for 48 h. **c**, **e** Combination index (CI) plot for anisomycin and apicidin in SK-N-DZ (**c**) and SK-N-BE(2)-C (**e**). CI values are plotted as a function of the fractional inhibition of cell viability by computer simulation using CompuSyn, where the gray circles represent the actual experimental points. CI values: synergism (CI < 0.9), additive effect (CI = 0.9–1.1), and antagonism (CI > 1.1), and the Fa value indicates cell fraction affection by the combination of anisomycin and apicidin. The mean CI and Fa values are listed in the table (**c**, **e**). Three biological replicates were performed with two technical replicates each. Data depicted are the mean ± SEM

Anti-metabolic agents have been used in cancer treatment for decades, however, a relatively low number of metabolic inhibitors developed for cancer therapy due to the adverse effects on patients by blocking the synthesis of vital cellular constituents from metabolic pathways^41^. Our strategy for identifying anti-cancer agents is to screen for compounds that particularly target MYCN-regulated metabolic genes across cell lines by using transcriptomic profiles^31,32^. By matching gene signatures with drug signatures, we systematically identified the gene expressions of *MTHFD2* and *PAICS* were significantly suppressed by anisomycin and apicidin across ten cell lines (Fig. 7a and Supplementary Fig. 9). Anisomycin is a bacterial antibiotic which inhibits protein biosynthesis by blocking peptide bond formation in ribosomes, and activates kinase cascades including JNK, p38 MAPK signaling pathways^42–44^. Apicidin is a fungal metabolites shown to inhibit histone deacetylase, and induce p21(WAD1/Cip1), caspase-3 and caspase 9, and DNA fragmentation^45,46^.

**Fig. 8:**
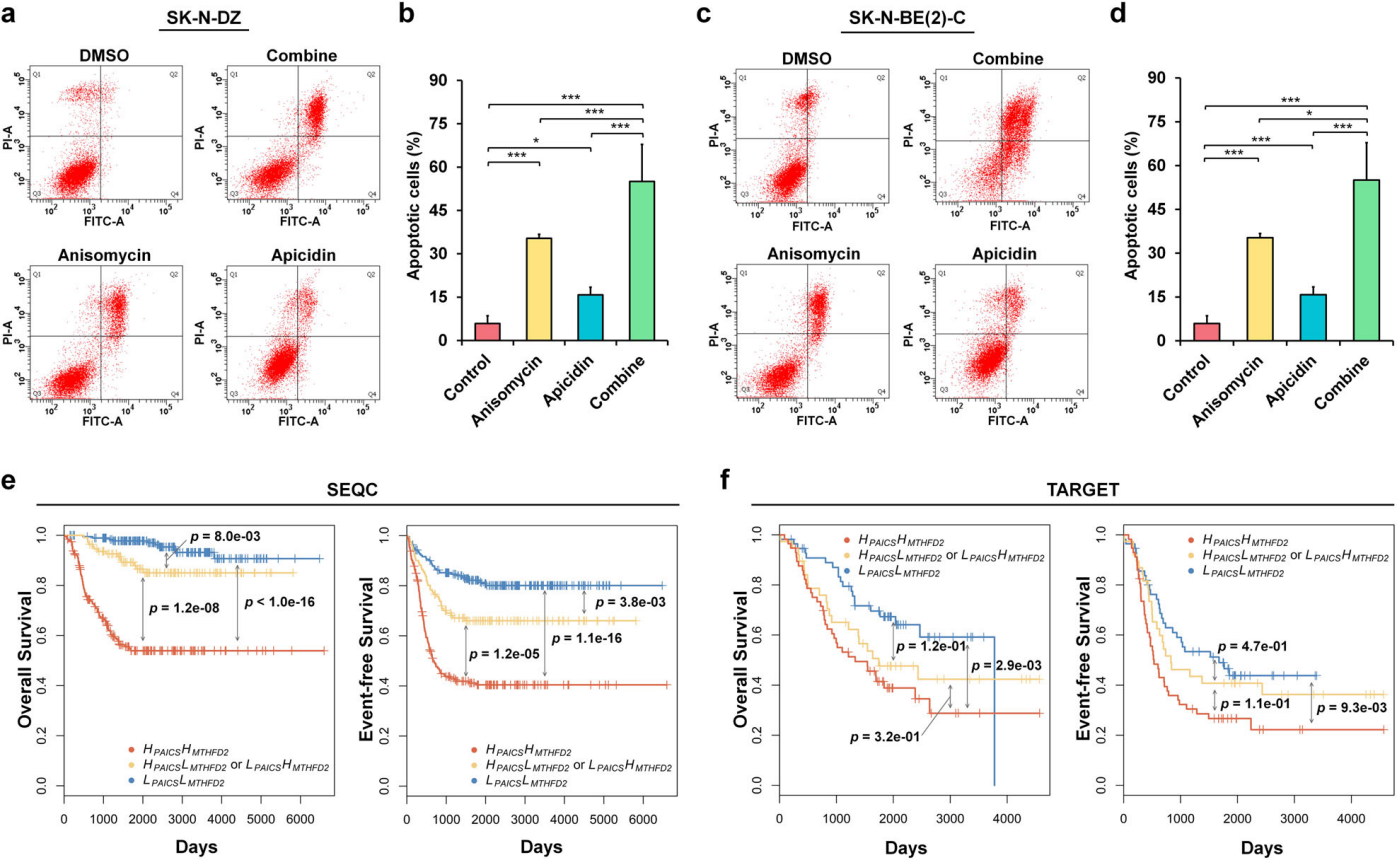
Combinatorial targeting of *MTHFD2* and *PAICS* induces cell apoptosis, and a low-low expression of *MTHFD2* and *PAICS* suppresses patient survival in neuroblastoma. **a**–**d** Apoptotic cell death was measured by flow cytometry for drug combination assay. **a**, **c** Representative flow cytometry profiles using Annexin V-FITC/PI staining for apoptosis in SK-N-DZ (**a**) and SK-N-BE(2)-C (**c**) cells. **b**, **d** Quantification of apoptotic cells between anisomycin, apicidin, and the combined treatment in SK-N-DZ (**b**) and SK-N-BE(2)-C (**d**) cells. Experiments are performed in triplicate and data are expressed as the mean ± SEM. *P < 0.05; **P < 0.01; ***P < 0.001. **e**, **f** Kaplan-Meier survival curves of overall and event-free survival of patients with neuroblastoma. Neuroblastoma patients with high-high expression of *MTHDF2* and *PAICS* (H_*MTHFD2*_H_*PAICS*_); low-low expression of *MTHDF2* and *PAICS* (L_*MTHFD2*_L_*PAICS*_), and high-low or low-high expression of *MTHFD2* and *PAICS* (H_*MTHFD2*_L_*PAICS*_ or L_*MTHFD2*_H_*PAICS*_) from SEQC (**e**) and TARGET (**f**) neuroblastoma cohorts. The median of the gene expression was used as the cutoff to straitify patients into high and low groups. The differences in survival time between groups were assessed by the log-rank test

To investigate the effects of anisomycin combined with apicidin on MNA neuroblastoma, drug combination assays were performed using SK-N-DZ and SK-N-BE(2)-C cells with various concentrations for 24 and 48 h (Fig. 7b–e and Supplementary Fig.10). The drug combination assay showed synergistic effects on the inhibition of MNA neuroblastoma cell proliferation, as compared with either anisomycin or apicidin treatment alone (Fig. 7b, d). Furthermore, the mean CI values were 0.71 and 0.74 in SK-N-DZ and SK-N-BE(2)-C, respectively, which indicated synergistic drug combinations (Fig. 7c, e). In addition, the non-MNA SK-N-AS and SK-N-SH neuroblastoma cells displayed a less sensitive effects to the anisomycin and apicidin treatments (Supplementary Fig. 11). Anisomycin and apicidin have been reported to induce apoptosis in certain cell types^46,47^, however, the effects of drug combination on apoptosis in neuroblastoma remains unidentified. Therefore, we performed apoptosis assays and found that both anisomycin and apicidin treatments induced apoptosis in MNA neuroblastoma (Fig. 8a–d). Notably, the co-treatment results exhibited the most significant cell apoptosis (Fig. 8b, d).

Next, we examined the *MTHFD2/PAICS* gene combinations with synergistic effects on neuroblastoma patient survival. First, the median expression value of the given gene was used to define the high and low expressions, and then we split neuroblastoma patients into three groups: high-high expression of *MTHDF2* and *PAICS* (H_*MTHFD2*_H_*PAICS*_); low-low expression of *MTHDF2* and *PAICS* (L_*MTHFD2*_L_*PAICS*_), and high-low or low-high expression of *MTHFD2* and *PAICS* (H_*MTHFD2*_L_*PAICS*_ or L_*MTHFD2*_H_*PAICS*_). Interestingly, the H_*MTHFD2*_H_*PAICS*_ group had the worst overall and event-free survival against the other groups, and the H_*MTHFD2*_L_*PAICS*_ or L_*MTHFD2*_H_*PAICS*_ group had poorer survival than L_*MTHFD2*_L_*PAICS*_, but better than the H_*MTHFD2*_H_*PAICS*_ (Fig. 8e, f). Altogether, the patient survival yielded consistent results as the drug combination assays that simultaneously suppressing *MTHFD2* and *PAICS* expressions showed a synergism in MNA neuroblastoma patients.

## Discussion

MYCN serves as an oncogenic regulator that mediates aberrant signaling pathways and leads to HR neuroblastoma. Recent studies achieved some clinical success to indirectly target MYCN, since developing direct MYCN-target agent encounters difficulties and has not yet been established^38,48,49^. Here, we addressed highly confident MYCN-targeted genes which may provide unbiased and genome-wide mapping of MYCN-gene interactions. Altogether, we revealed 427 potential MYCN-targeted genes, contributing to characterize downstream signaling pathways.

Cancer metabolism is a complex enzyme-catalyzed reaction that involves multiple factors to overcome any single disruption^50^. Thus, we aim to investigate the MYCN-regulated interconnection of metabolic pathways and biological processes. The potential MYCN-targeted genes are found to be involved in purine biosynthesis which consists ten steps and six enzymes from phosphoribosyl pyrophosphate (PRPP) to inosine monophosphate (IMP), guanosine monophosphate (GMP), and adenosine monophosphate (AMP)^51^. Purinosome was coined to describe the reversible six-enzyme complex for purine biosynthesis that assembled and co-localized on mitochondria while the purine levels of cytosol depleted^23,52^. Besides, one carbon pool by folate was also identified to be associated with MNA neuroblastoma, which generated formates, the precursor to 10-formyltetrahydrofolate in the mitochondria and exported into cytosol for the need of transformylase enzymes, GRAT and ATIC in purine biosynthesis^21^. On the other hand, loss of MTHFD2 would cause folate trapping within the mitochondria, therefore, folate-dependent pathways may be altered^24,53,54^.

Previous studies indicate that amino acid metabolism, nucleic acid metabolism, and mitochondrial metabolism play important roles in cancer progression^55,56^. Biosynthetic pathways are essential for cancer metabolism since they generate energy and macromolecules that are required for replicative cell division and tumor growth^56^. Our targeted metabolomics analysis indicated that the levels of IMP and GMP were significantly higher in MNA neuroblastoma compared to non-MNA neuroblastoma cells, suggesting MNA neuroblastoma may require higher demand in purine biosynthesis for rapid cell proliferation. The MYCN-targeted gene pair results implied that MYCN could regulate the interconnection of metabolic pathways by MTHFD2 and PAICS in two directions. MYCN transcriptionally regulates the mitochondrial metabolic enzyme MTHFD2 for formate exportation and the bifunctional enzyme PAICS of the purinosome to generate SAICAR for purine biosynthesis.

Treatment that combines two or more specific therapeutic targets might reduce the dosage and side-effect for aggressive therapy in children^57^. Our dual knockdown and co-treatment assays that target MTHFD2 and PAICS had synergistic effects on MNA neuroblastoma which might diminish the aggressiveness and tumor progression ability. In conclusion, establishing MYCN downstream signaling pathways in neuroblastoma may improve our capacity in identifying molecular interactions, disease pathways, and even targeted drug discovery.

## Supplementary information

Author Contribution Form

Supplementary Information

Supplementary Table S1

Supplementary Table S2

Supplementary Table S3

Supplementary Table S4

Supplementary Table S5

Supplementary figure legends
