# Supplementary material for: Combinatorial targeting of MTHFD2 and PAICS in purine synthesis as a novel therapeutic strategy
Source: Cell Death Dis. 2019 Oct 17;10(11):786. doi: 10.1038/s41419-019-2033-z (PMC6797810; doi:10.1038/s41419-019-2033-z)
Supplement: Supplementary file 1 — Author Contribution Form [file 41419_2019_2033_MOESM1_ESM.pdf]

DECLARATION OF CONTRIBUTIONS TO ARTICLE

ADMC

Manuscript Number: CDDIS-19-1779-T

Journal Name: *Cell Death & Disease* (the 'Journal')

Proposed Title of the Contribution:

Combinatorial targeting of MTHFD2 and PAICS in purine synthesis as a novel therapeutic strategy (the 'Contribution)

Author(s):

Chantal Hoi Yin Cheung, Chia-Lang Hsu, Chao-Yin Tsuei, Tzu-Ting Kuo, Chen-Tsung Huang, Wen-Ming Hsu, Yun-Hsien Chung, Hsin-Yi Wu, Cheng-Chih Hsu, Hsuan-Cheng Huang, and Hsueh-Fen Juan. (the 'Authors')

For all *CDDis* articles, each person named as an author in the published version must be able to show he or she has contributed substantially to the article.

Authorship credit should be based on 1) substantial contributions to conception and design, acquisition of data, or analysis and interpretation of data; 2) drafting the article or revising it critically for important intellectual content; and 3) final approval of the version to be published. Authors should meet conditions 1, 2 and 3.

Any person who cannot be shown to have made a substantial contribution to the article cannot be listed as an author in the final version. The name of any person who is deemed to have made a minor contribution can, however, appear in the Acknowledgments section of the article.

Please complete the table below to indicate the contributions of all named authors to the manuscript.

Author Full Name:	Specification of Contribution to the Manuscript:
Chantal Hoi Yin Cheung	conceptualized and designed research, developed the methodology, administrative, technical or material supported including reporting or organizing data, constructing databases, analyzed and interpreted the data including statistical analysis, biostatistics, computational analysis, wrote, reviewed and/or revised the manuscript.
Chia-Lang Hsu	analyzed and interpreted the data including statistical analysis, biostatistics, computational analysis, wrote, reviewed and/or revised the manuscript.
Chao-Yin Tsuei	analyzed and interpreted the data including statistical analysis, biostatistics, computational analysis.
Tzu-Ting Kuo	analyzed and interpreted the data including statistical analysis, biostatistics, computational analysis.
Chen-Tsung Huang	analyzed and interpreted the data including statistical analysis, biostatistics, computational analysis.
Wen-Ming Hsu	acquired the data including animals, managed patients, and provided facilities.
Yun-Hsien Chung	analyzed and interpreted the data including statistical analysis, biostatistics, computational analysis.
Hsin-Yi Wu	acquired the data including animals, managed patients, and provided facilities.
Cheng-Chih Hsu	acquired the data including animals, managed patients, and provided facilities.
Hsuan-Cheng Huang	conceptualized and designed research, developed the methodology, administrative, technical or material supported including reporting or organizing data, constructing databases, wrote, reviewed and/or revised the manuscript and supervised this project.
Hsueh-Fen Juan	conceptualized and designed research, developed the methodology, administrative, technical or material supported including reporting or organizing data, constructing databases, wrote, reviewed and/or revised the manuscript and supervised this project.

Please complete the table below to indicate the contributions of all named authors to the figures.

Figure 1:

Chantal Hoi Yin Cheung
Chia-Lang Hsu
Chen-Tsung Huang
Hsin-Yi Wu
Cheng-Chih Hsu
Hsuan-Cheng Huang
Hsueh-Fen Juan

Figure 2:

Chao-Yin Tsuei
Tzu-Ting Kuo
Wen-Ming Hsu
Hsuan-Cheng Huang
Hsueh-Fen Juan

Figure 3:

Chao-Yin Tsuei
Tzu-Ting Kuo
Hsuan-Cheng Huang
Hsueh-Fen Juan

Figure 4:

Chantal Hoi Yin Cheung
Hsin-Yi Wu
Cheng-Chih Hsu
Hsuan-Cheng Huang
Hsueh-Fen Juan

Figure 5:

Chantal Hoi Yin Cheung
Hsuan-Cheng Huang
Hsueh-Fen Juan

Figure 6:

Figure 6
Chantal Hoi Yin Cheung
Hsuan-Cheng Huang
Hsueh-Fen Juan
Figure 7
Chen-Tsung Huang
Yun-Hsien Chung
Hsuan-Cheng Huang
Hsueh-Fen Juan
Figure 8
Chantal Hoi Yin Cheung
Hsuan-Cheng Huang
Hsueh-Fen Juan

Signed for and on behalf of the Author(s):	Print Name:	Date:
Hsueh-Fen Juan	Hsueh-Fen Juan	August 12, 2019
