# Supplementary material for: Combinatorial targeting of MTHFD2 and PAICS in purine synthesis as a novel therapeutic strategy
Source: Cell Death Dis. 2019 Oct 17;10(11):786. doi: 10.1038/s41419-019-2033-z (PMC6797810; doi:10.1038/s41419-019-2033-z)
Supplement: Supplementary file 2 — Supplementary Information [file 41419_2019_2033_MOESM2_ESM.docx]

**Supplementary Information**

**Combinatorial targeting of MTHFD2 and PAICS in purine synthesis as a novel therapeutic strategy**

Chantal Hoi Yin Cheung^1^, Chia-Lang Hsu^1,2^, Chao-Yin Tsuei^3^, Tzu-Ting Kuo^3^, Chen-Tsung Huang^4^, Wen-Ming Hsu^5^, Yun-Hsien Chung^1^, Hsin-Yi Wu^6^, Cheng-Chih Hsu^6^, Hsuan-Cheng Huang^7*^, and Hsueh-Fen Juan^1,3,4*^

^1^Department of Life Science, National Taiwan University, Taipei 10617, Taiwan.

^2^Department of Medical Research, National Taiwan University Hospital, Taipei 10002, Taiwan

^3^Institute of Molecular and Cellular Biology, National Taiwan University, Taipei 10617, Taiwan.

^4^ Graduate Institute of Biomedical Electronics and Bioinformatics, National Taiwan University, Taipei 10617, Taiwan.

^5^Department of Surgery, National Taiwan University Hospital and College of Medicine National Taiwan University, Taipei 10017, Taiwan.

^6^Department of Chemistry, National Taiwan University, Taipei, Taiwan.

^7^Institute of Biomedical Informatics, National Yang-Ming University, Taipei 11221, Taiwan.

**Summary:**

**The Supplementary Information contains two sections:**

**I. Supplementary Figures**

- Supplementary Fig. S1. Mass spectra of the standards, SK-N-AS and SK-N-DZ.
- Supplementary Fig. S2*. MTHFD2* and *PAICS* are correlated with *MYCN* status.
- Supplementary Fig. S3. Full length western blots of six neuroblastoma cell lines.
- Supplementary Fig. S4. *MTHFD2* and *PAICS* anti-MYCN ChIP-seq profiles in *MYCN* amplified neuroblastoma cell.
- Supplementary Fig. S5. Immunoblotting images of SK-N-AS, SK-N-DZ, and stable SK-N-DZ cells of shLacZ, shMTHFD2, shPAICS, and shMTHFD2/PAICS
- Supplementary Fig. S6. Mass spectra of stable knock-down cell lines.
- Supplementary Fig. S7. Immunoblotting images of SK-N-DZ and stable SK-N-DZ cells.
- Supplementary Fig. S8. Cell viability of single knockdown of MTHFD and PAICS in SK-N-DZ cells.
- Supplementary Fig. S9. The structure and summary of the compounds used in drug combination assay.
- Supplementary Fig. S10. Percent cell growth of MNA neuroblastoma cells for anisomycin, apicidin, or combined treatment at indicated dosage.
- Supplementary Fig. S11. Percent cell growth of non-MNA neuroblastoma cells for anisomycin, apicidin, or combined treatment at indicated dosage.

**II. Supplementary Tables (separate Microsoft Excel files)**

- Supplementary Table S1. qRT-PCR primer sequences.
- Supplementary Table S2. Promotor assay primer sequences.
- Supplementary Table S3. shRNA target sequences.
- Supplementary Table S4. Differentially expressed genes with MYCN bound.
- Supplementary Table S5. KEGG enrichment analysis results.

**I. Supplementary Figures**

**Supplementary Fig. S1**

**
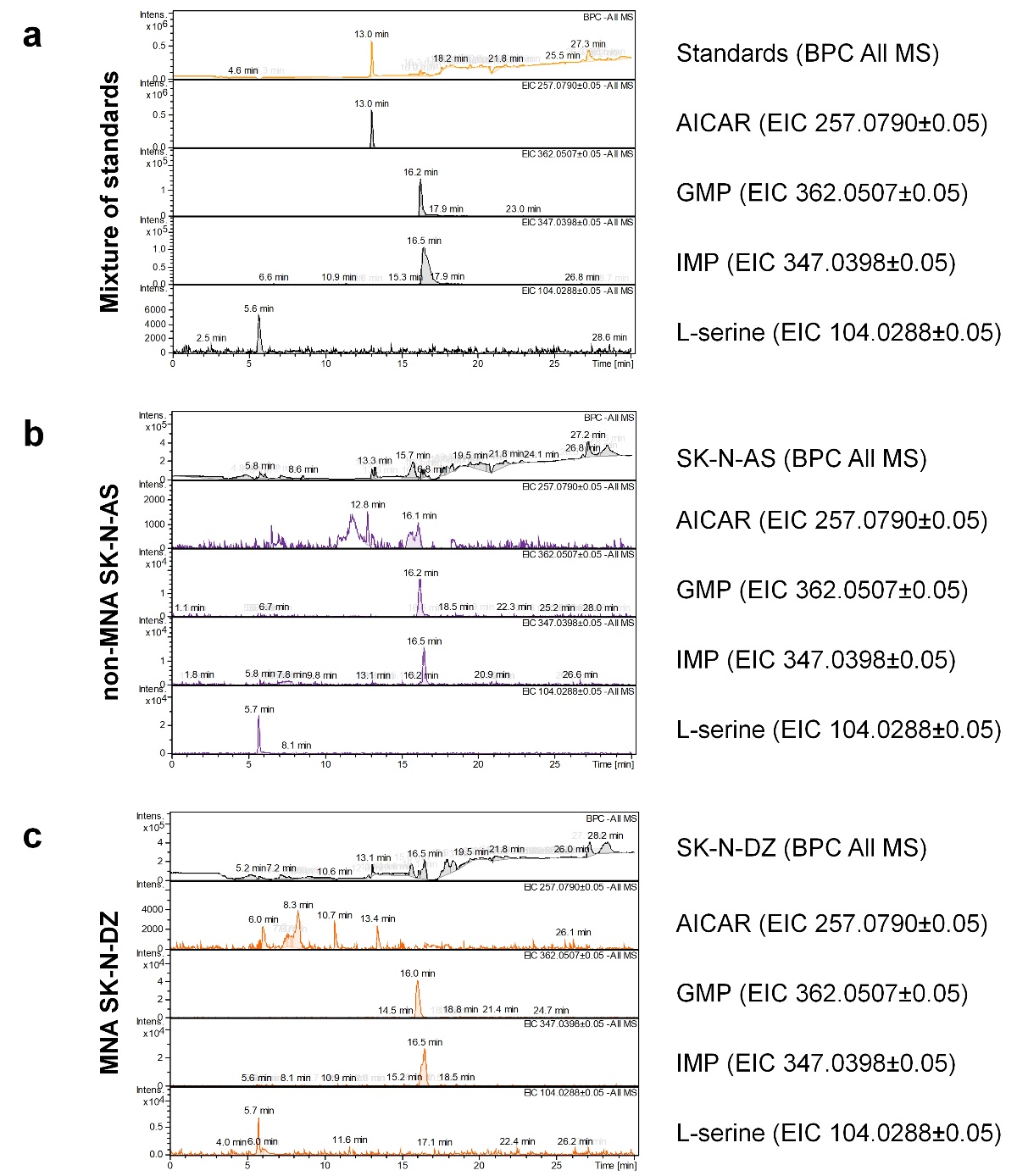
**

**Supplementary Fig. S2**

**
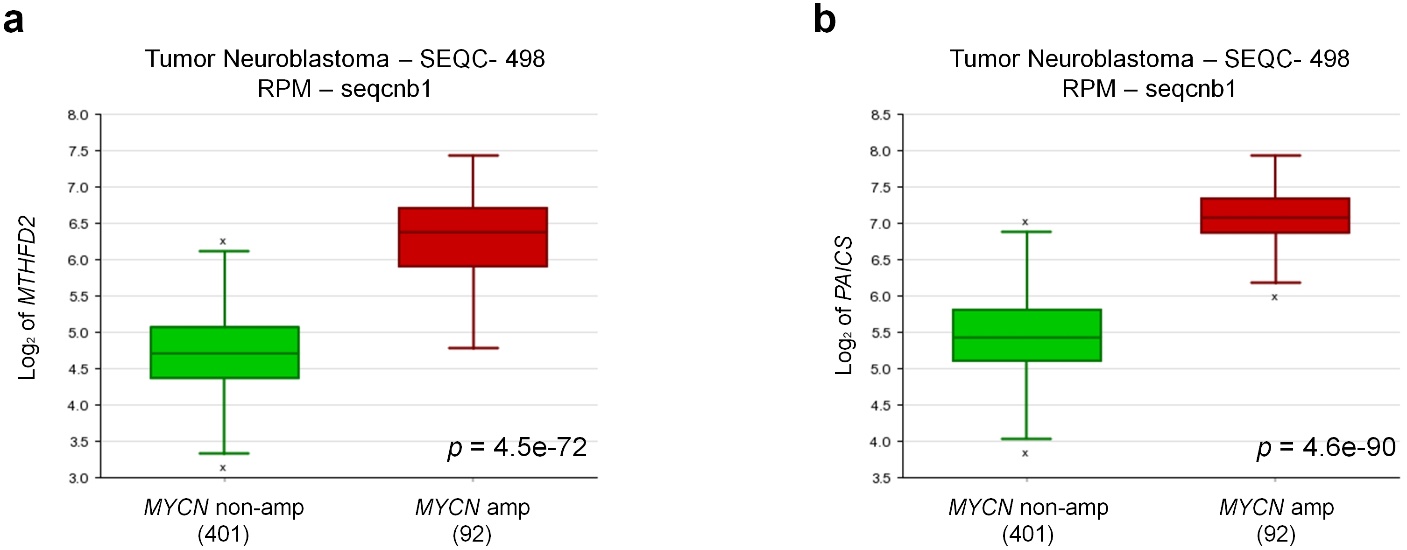
**

**Supplementary Fig. S3**


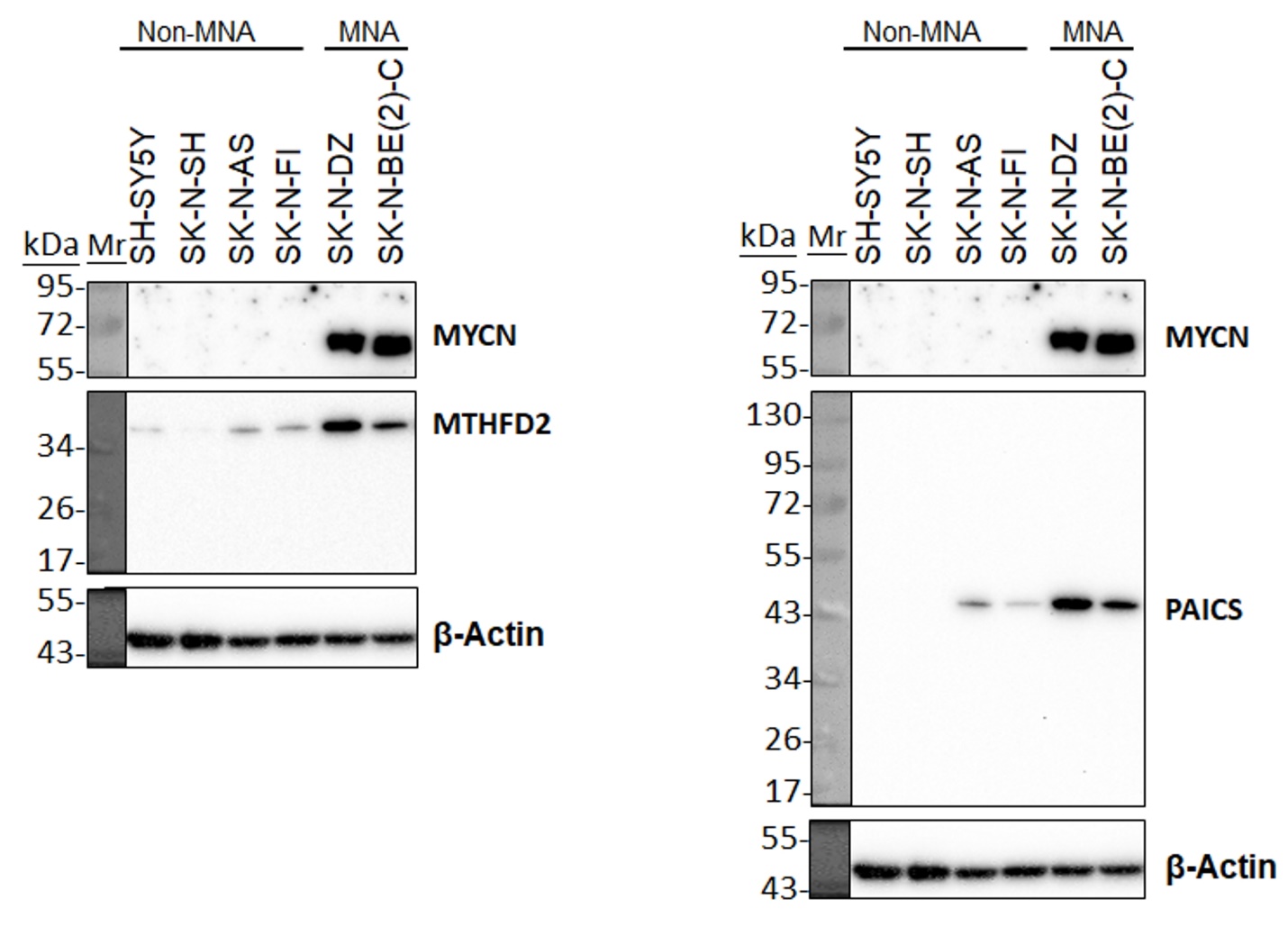


**Supplementary Fig. S4**

**
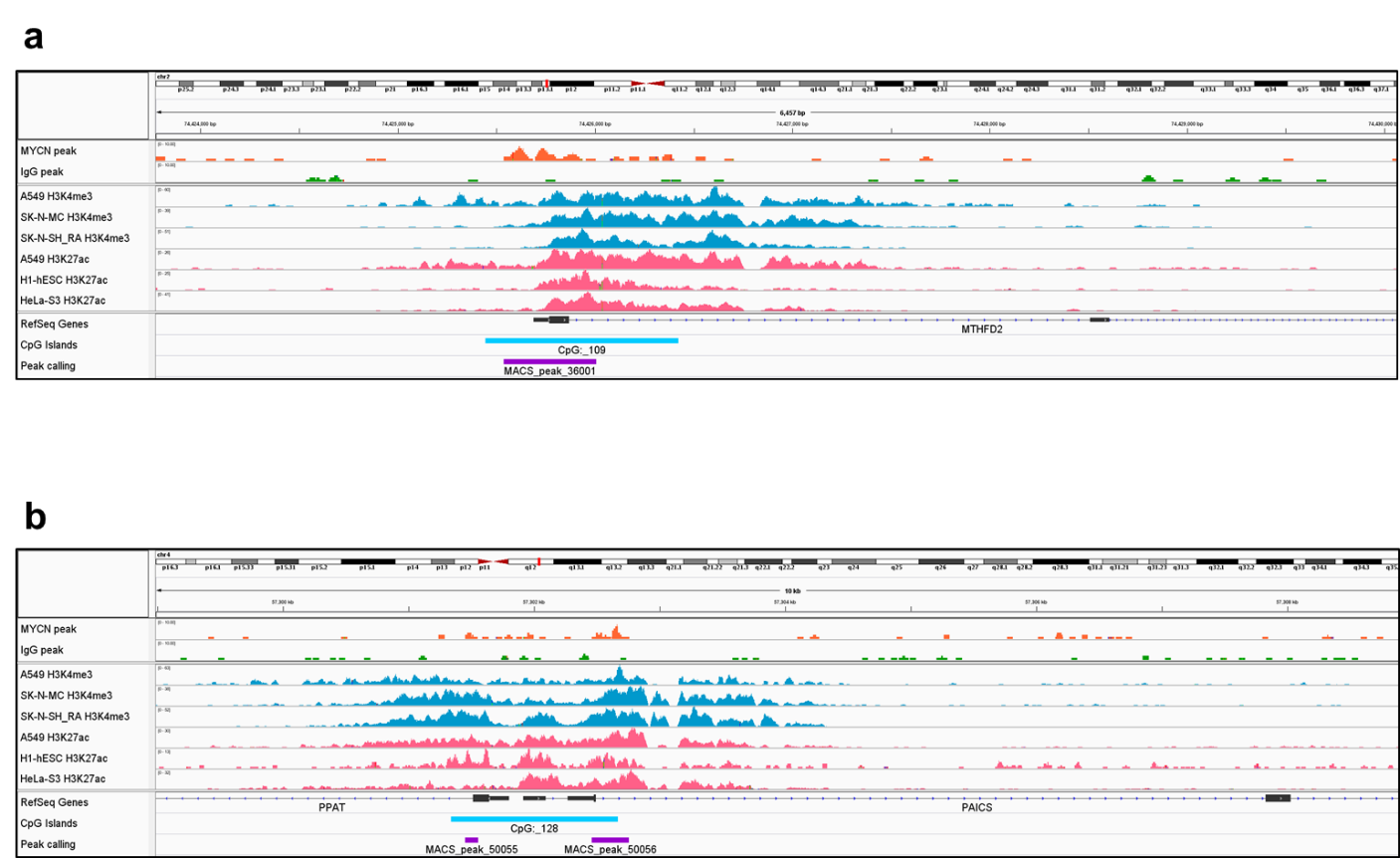
**

**Supplementary Fig. S5**

**
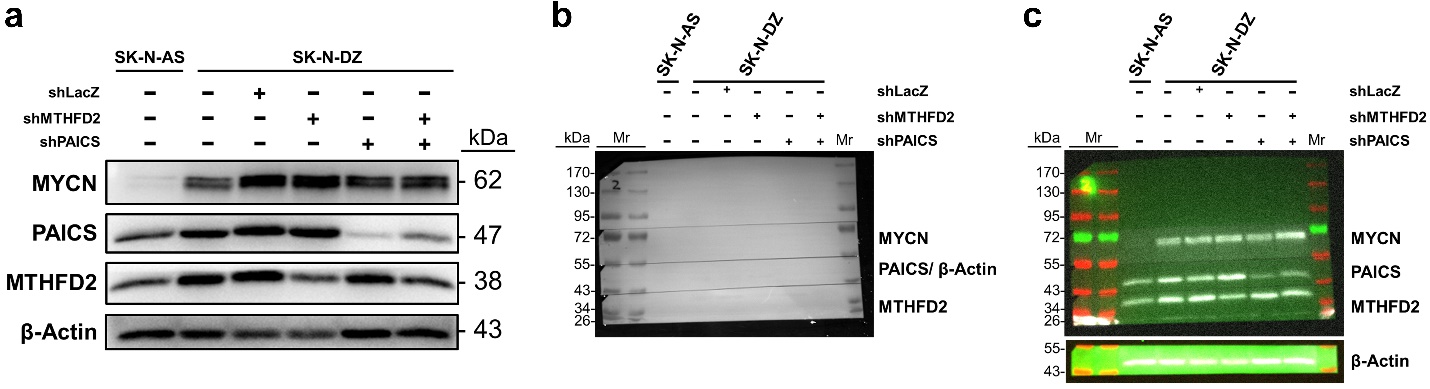
**

**Supplementary Fig. S6**

**
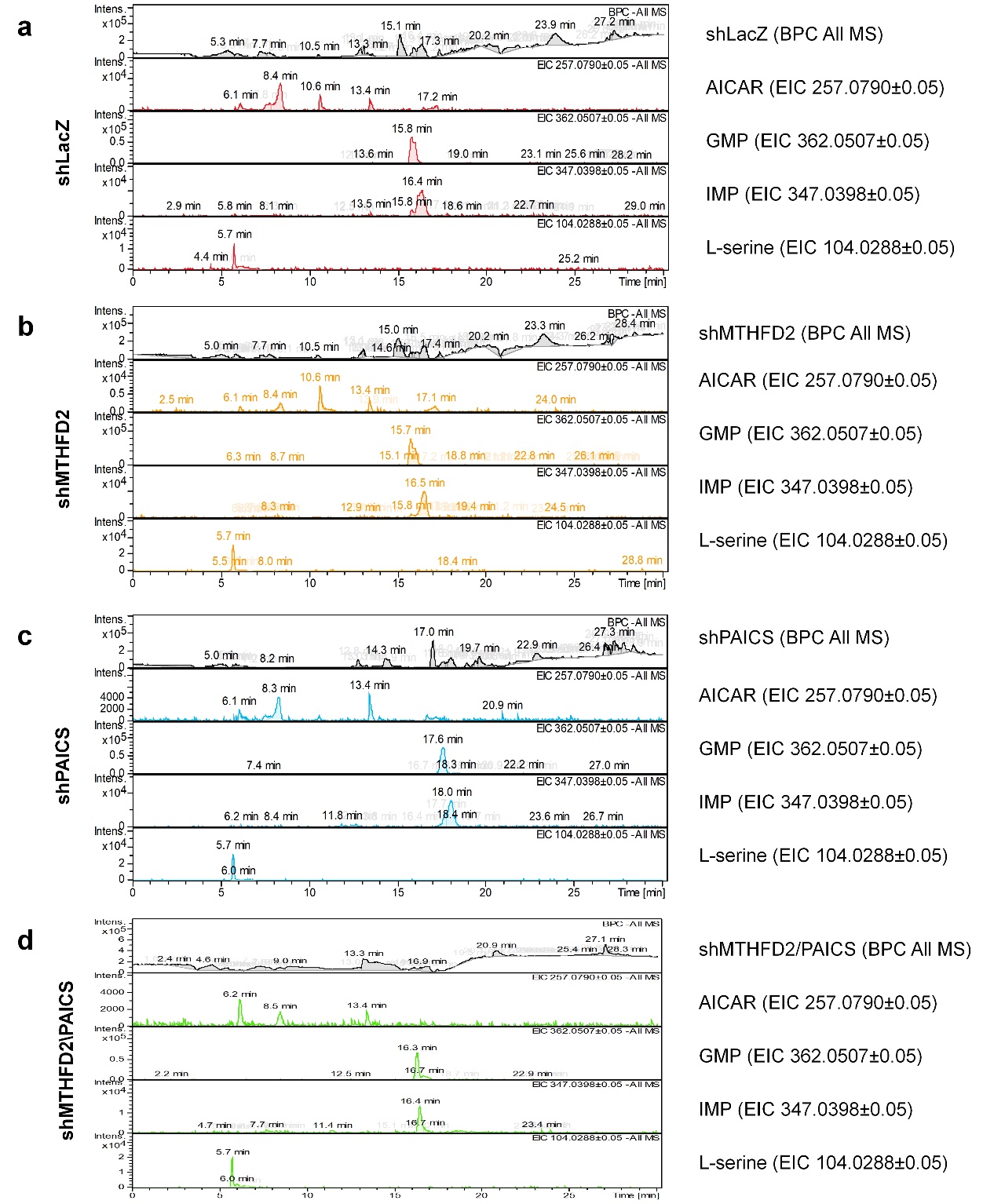
**

**Supplementary Fig. S7**


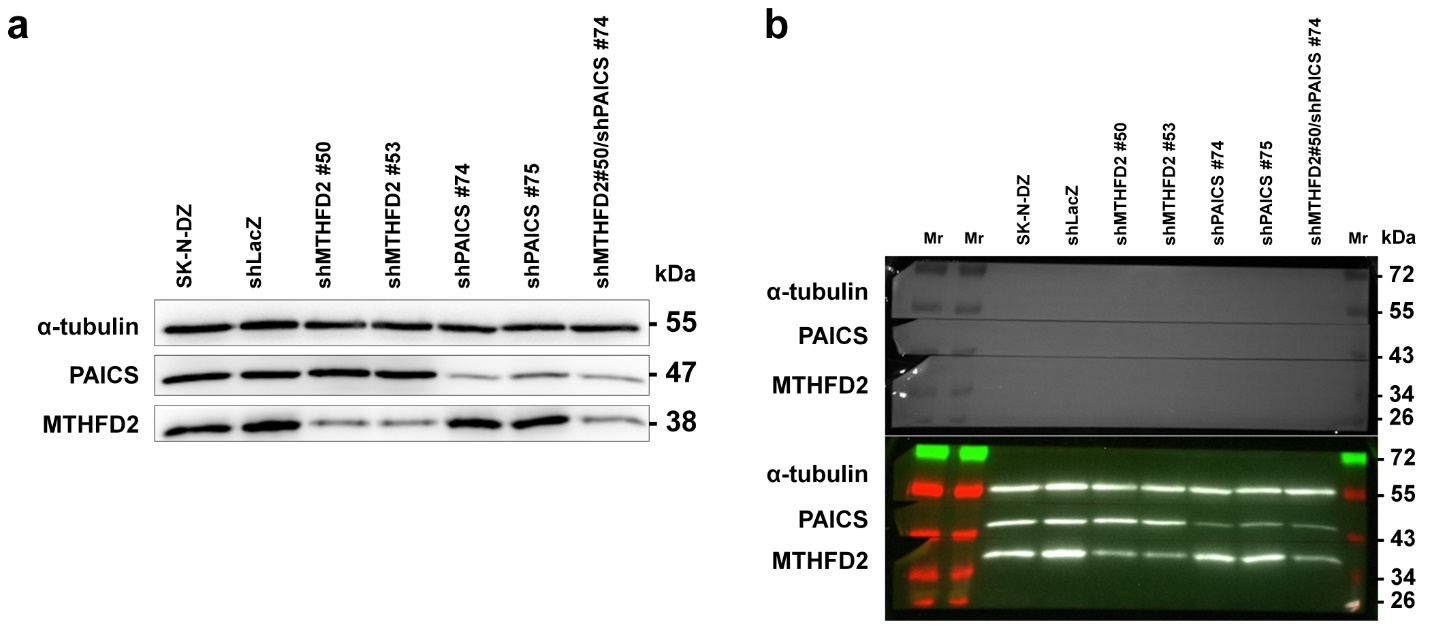


**Supplementary Fig. S8**


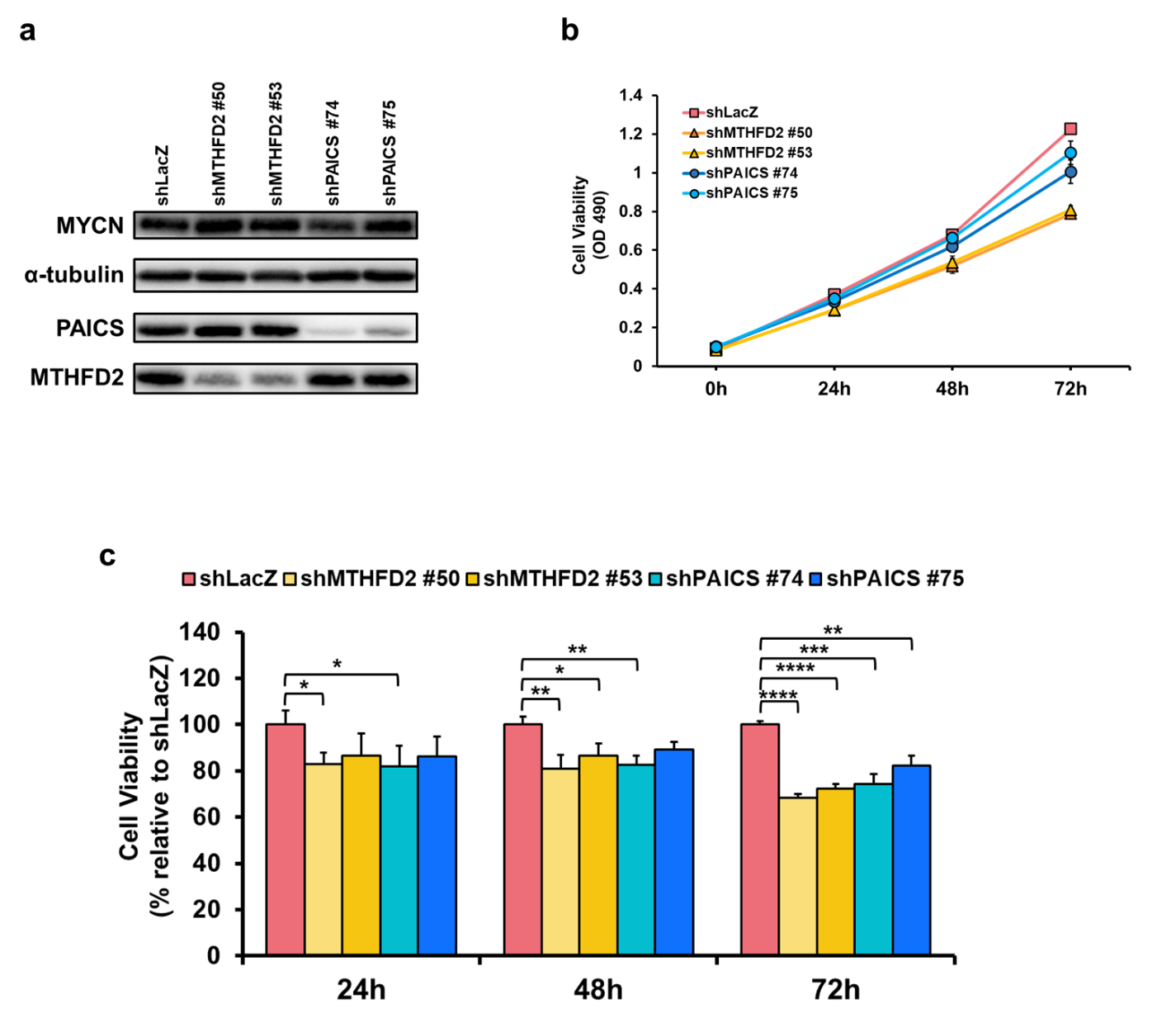


**Supplementary Fig. S9**


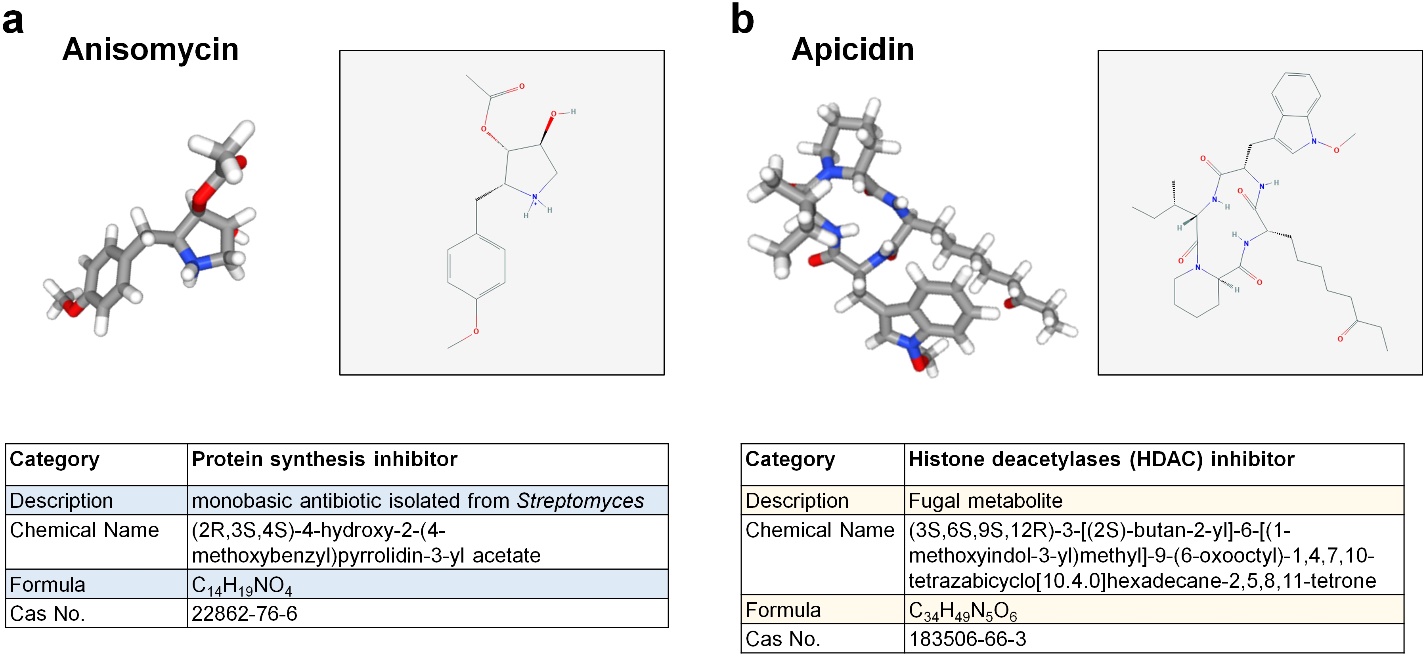


**Supplementary Fig. S10**

**
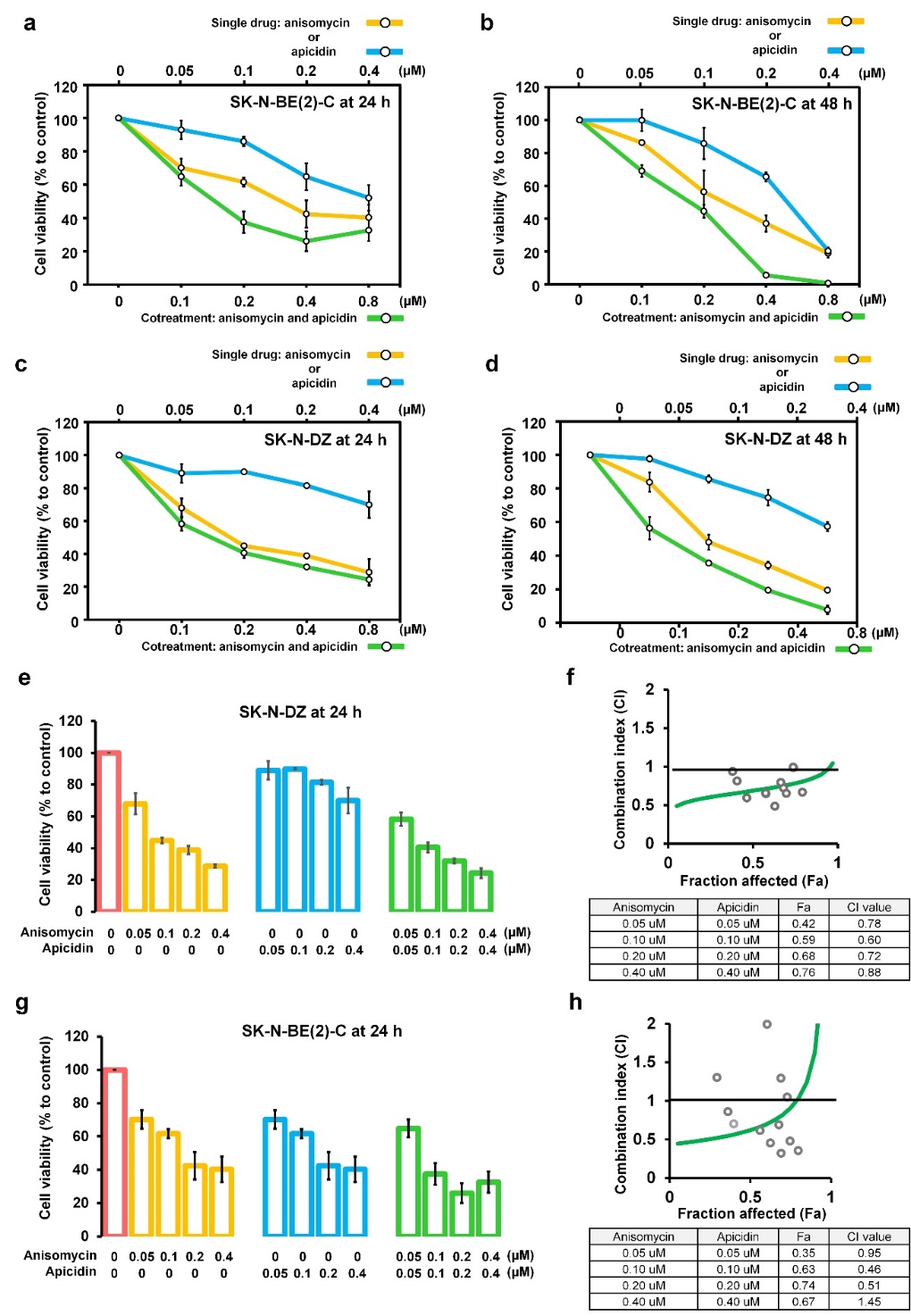
**

**Supplementary Fig. S11**

**
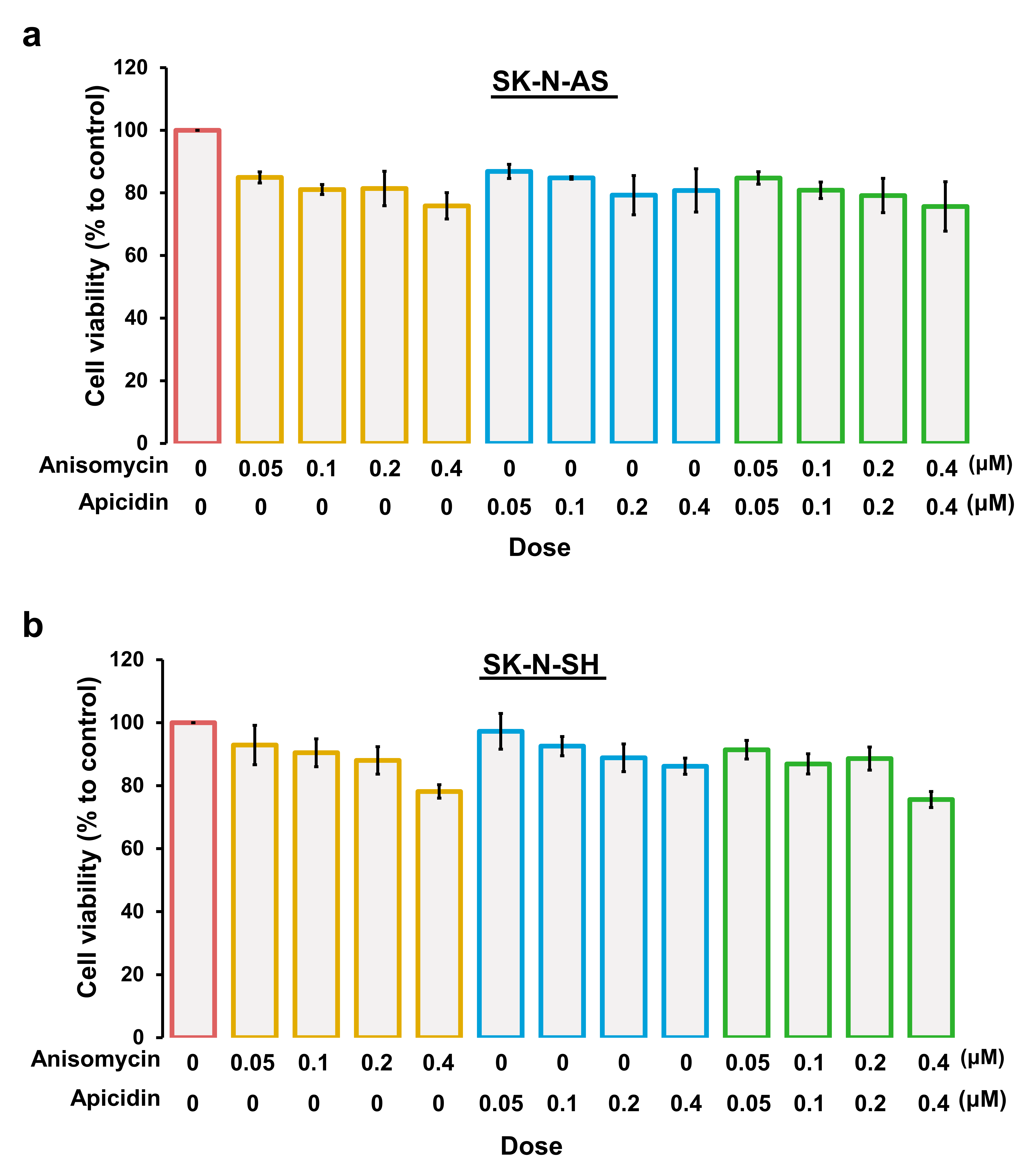
**
