# Supplementary material for: Combinatorial targeting of MTHFD2 and PAICS in purine synthesis as a novel therapeutic strategy
Source: Cell Death Dis. 2019 Oct 17;10(11):786. doi: 10.1038/s41419-019-2033-z (PMC6797810; doi:10.1038/s41419-019-2033-z)
Supplement: Supplementary file 8 — Supplementary figure legends [file 41419_2019_2033_MOESM8_ESM.docx]

**Supplementary Fig. S1.** **Mass spectra of the standards, SK-N-AS and SK-N-DZ.** Mass spectra in the range m/z 50–500 were obtained by using electrospray ionization in negative-ion mode**.** A mixture of L-serine (*m*/*z* 104.0288), AICAR (*m*/*z* 257.0790), IMP (*m*/*z* 347.0398), and GMP (*m*/*z* 362.0507) standards **(a)**, non-MNA SK-N-AS **(b)**, and MNA SK-N-DZ **(c)** neuroblastoma cell lines were analyzed.

**Supplementary Fig. S2. *MTHFD2* and *PAICS* are correlated with *MYCN* status.** Box plots of *MTHFD2* **(a)** and *PAICS* **(b)** expressions in non-MNA and MNA neuroblastoma. The analysis was performed in R2 platform (http://r2.amc.nl/) using SEQC-NB498 dataset. *r*: Pearson correlation coefficient.

**Supplementary Fig. S3. Full length western blots of six neuroblastoma cell lines.**

These full length blots correspond to the cropped images presented in Figure 2.

**Supplementary Fig. S4. *MTHFD2* (a) and *PAICS* (b) anti-MYCN ChIP-seq profiles in *MYCN* amplified neuroblastoma cell.** The data is visulaized by Integrative Genomics Viewer (IGV) which was extracted from our previous published anti-MYCN ChIP-seq.

**Supplementary Fig. S5.** **Immunoblotting images of SK-N-AS, SK-N-DZ, and stable SK-N-DZ cells of shLacZ, shMTHFD2, shPAICS, and shMTHFD2/PAICS.** Immunoblot analysis of MYCN, PAICS, MTHFD2, and β-Actin protein levels (**a**), bright field (**b**) and merged with marker images (**c**) of SK-N-AS, SK-N-DZ, and stable SK-N-DZ cells of shLacZ, shMTHFD2, shPAICS, and shMTHFD2/PAICS.

**Supplementary Fig. S6. Mass spectra of stable knock-down cell lines.** Mass spectra in the range m/z 50–500 were obtained by using electrospray ionization in negative-ion mode. Stable knock-down of MNA SK-N-DZ neuroblastoma cell lines: shLacZ (**a**), shMTHFD2 (**b**), shPAICS (**c**), shMTHFD2/PAICS (**d**) were analyzed for the abundance of L-serine (*m*/*z* 104.0288), AICAR (*m*/*z* 257.0790), IMP (*m*/*z* 347.0398), and GMP (*m*/*z* 362.0507).

**Supplementary Fig. S7.** **Immunoblotting images of SK-N-DZ and stable SK-N-DZ cells.** Immunoblot analysis of α-tubulin, PAICS, MTHFD2 protein levels (**a**), bright field (**b, upper**) and merged with marker images (**b, bottom**) of SK-N-DZ, and stable shLacZ, shMTHFD2 #50 and #53, shPAICS #74 and #75, and shMTHFD2#50/PAICS#74 SK-N-DZ cells.

**Supplementary Fig. S8. Cell viability of single knockdown of MTHFD and PAICS in SK-N-DZ cells. (a)** Immunoblotting images of shLacZ, shMTHFD2 (#50 and #53), shPAICS (#74 and #75) SK-N-DZ cells. **(b, c)** Cell proliferation was affected by knockdown of either MTHFD2 or PAICS.

**Supplementary Fig. S9. The structure and summary of the compounds used in drug combination assay.** The 2D and 3D structures of the Anisomycin (a) and Apicidin (b) were downloaded from PubChem database (https://pubchem.ncbi.nlm.nih.gov).

**Supplementary Fig. S10. Percent cell growth of MNA neuroblastoma cells for anisomycin, apicidin, or combined treatment at indicated dosage.** Percent cell growth of MNA SK-N-DZ (**a, b**) and SK-N-BE (2)-C (**c, d**) cells relative to corresponding controls treated with anisomycin, apicidin, or the combination at indicated dosage for 24 h and 48 h. The bar plot reveals the cell viability of the indicated drug combination for 24 h (**e, g**). Combination Index (CI) was calculated by CompuSyn (http://www.combosyn.com); the grey circles are the actual doses, fraction affected (Fa) and CI values for 24 h. CI < 0.9 indicates that the interaction of the two drugs is synergistic, and the Fa value indicates cell fraction affection by the combination of anisomycin and apicidin; the mean Fa and CI values are listed in the table (**f, h**). Three biological replicates were performed with two technical replicates each. Data depicted are the mean ± SEM.

**Supplementary Fig. S11. Percent cell growth of non-MNA neuroblastoma cells for anisomycin, apicidin, or combined treatment at indicated dosage.** Percent cell growth of MNA SK-N-AS (**a**) and SK-N-SH (**b**) cells relative to corresponding controls treated with anisomycin, apicidin, or the combination at indicated dosage for 24 h. The bar plot reveals the cell viability of the indicated drug combination. Three biological replicates were performed with four technical replicates each. Data depicted are the mean ± SEM.
